# The Meningococcal Cysteine Transport System Plays a Crucial Role in *Neisseria meningitidis* Survival in Human Brain Microvascular Endothelial Cells

**DOI:** 10.1128/mBio.02332-18

**Published:** 2018-12-11

**Authors:** Hideyuki Takahashi, Haruo Watanabe, Kwang Sik Kim, Shigeyuki Yokoyama, Tatsuo Yanagisawa

**Affiliations:** a Department of Bacteriology I, National Institute of Infectious Diseases, Tokyo, Japan; b School of Medicine, International University of Health and Welfare, Chiba, Japan; c Division of Pediatric Infectious Diseases, Department of Pediatrics, School of Medicine, Johns Hopkins University, Baltimore, Maryland, USA; d RIKEN Structural Biology Laboratory, Yokohama, Japan; University of Minnesota Medical School; Emory University; University of Würzburg

**Keywords:** *Neisseria meningitidis*, blood, cerebrospinal fluid, cysteine, glutathione, internalization, intracellular growth, reactive oxygen species, serum, survival

## Abstract

While Neisseria meningitidis typically exists in an asymptomatic nasopharyngeal carriage state, it may cause potentially lethal diseases in humans, such as septicemia or meningitis, by invading deeper sites in the body. Since the nutrient compositions of human cells are not always conducive to meningococci, N. meningitidis needs to exploit nutrients from host environments. In the present study, the utilization of cysteine by the meningococcal cysteine transport system (CTS) was analyzed for the pathogenesis of meningococcal infections. A N. meningitidis strain deficient in one of the three *cts* genes annotated as encoding cysteine-binding protein (*cbp*) exhibited approximately 100-fold less internalization into human brain microvascular endothelial cells (HBMEC) than the wild-type strain. This deficiency was restored by complementation with the three *cts* genes together, and the infectious phenotype of HBMEC internalization correlated with cysteine uptake activity. However, efficient accumulation of ezrin was observed beneath the *cbp* mutant. The intracellular survival of the *cbp* mutant in HBMEC was markedly reduced, whereas equivalent reductions of glutathione concentrations and of resistance to reactive oxygens species in the *cbp* mutant were not found. The *cbp* mutant grew well in complete medium but not in synthetic medium supplemented with less than 300 μM cysteine. Taking cysteine concentrations in human cells and other body fluids, including blood and cerebrospinal fluid, into consideration, the present results collectively suggest that the meningococcal CTS is crucial for the acquisition of cysteine from human cells and participates in meningococcal nutrient virulence.

## Abstract (precis)

Neisseria meningitidis colonizes at a nasopharynx of human as a unique host and has many strains that are auxotrophs for amino acids for their growth. To cause invasive meningococcal diseases (IMD) such as sepsis and meningitis, N. meningitidis passes through epithelial and endothelial barriers and infiltrates into blood and cerebrospinal fluid as well as epithelial and endothelial cells. However, meningococcal nutrients, including cysteine, become less abundant when it more deeply infiltrates the human body even during inflammation, such that N. meningitidis has to acquire nutrients in order to survive/persist, disseminate, and proliferate in humans. This was the first study to examine the relationship between meningococcal cysteine acquisition and the pathogenesis of meningococcal infections. The results of the present study provide insights into the mechanisms by which pathogens with auxotrophs acquire nutrients in hosts and may also contribute to the development of treatments and prevention strategies for IMD.

## INTRODUCTION

Neisseria meningitidis is a fastidious Gram-negative microorganism that generally exists in the noninvasive so-called “carrier state” at a rate of approximately 0.4% to 25% in human populations (1, 2). However, for reasons not yet fully understood, N. meningitidis exhibits the ability to cross the epithelial layer, infiltrate the bloodstream, evade the defenses of the human immune system, adhere to the endothelial layers of peripheral and brain vessels, cross the brain-blood barrier (BBB), and replicate in cerebrospinal fluid (CSF). While epidemiological analyses have suggested that a human genetic polymorphism (3) and environmental conditions such as smoking (4, 5) and climate conditions (6) are important for predicting the outcomes of infection, the reasons that IMD occurs in some individuals but not in others remain unclear.

Classical and recent advanced analyses such as reverse genetics have revealed so far that factors exposed to meningococcal surfaces such as the polysaccharide capsule, Opa and Opc (reviewed in references 7, 8, 9, and 10), NhhA (11), NadA (12), App (11), NalP (13), MspA (14), TspA (15), adhesion complex protein (ACP) (16), and fHbp (17) are related to the pathogenesis of the strain. However, the gene contents of meningococcal strains isolated from healthy carriers and patients with IMD were mostly indistinguishable (18), and genomic islands, which are found in pathogenic but not in nonpathogenic *Neisseria*, were not present in the meningococcal or neisserial genomes of any of the neisserial strains examined (19) or in meningococcal isolates from patients and healthy carriers (20, 21). Thus, the mechanisms by which N. meningitidis causes septicemia and meningitis in humans, in particular, the trigger to induce IMD from a carrier state, have not yet been elucidated (22, 23).

Meningococcal research has mainly focused on so-called “meningococcal virulence factors” that directly interact with the host components described above but do not involve microbial nutrients and metabolism in the host. However, acquisition of nutrients from human hosts appears to be important for meningococcal pathogenesis because the availability of the nutrient supply needed by facultative meningococci to cause IMD is limited in the human environment (24–26). A well-known example of the acquisition of nutrients is that of iron in humans; N. meningitidis robs human iron from host factors such as hemoglobin, transferrin, and lactoferrin (27). While human has as the first line of defense Fe sequestration from free Fe (called “nutrient immunity”) (28, 29), N. meningitidis overcomes the nutrient immunity by preparing bacterial high-affinity receptors to take iron from iron-chelating host molecules (29). Thus, metabolic adaptation in humans enables meningococci to exploit host resources, which supports the concept of bacterial “nutrient virulence” against hostile nutrient immunity as a crucial factor influencing invasive capabilities (25). Experimental data has indicated that many N. meningitidis strains are auxotrophs for at least 4 amino acids, namely, glutamate, cysteine, arginine, and glycine (30), and previous studies also demonstrated that glutamate uptake is required for bacterial intracellular growth/survival in human cells (31–33) as well as for survival and proliferation in mice (34, 35). Moreover, a reduced sulfur form of cysteine, cystine, was found to be necessary for meningococcal growth (36, 37), and many N. meningitidis strains had cysteine auxotroph for growth in the presence of low cysteine concentrations (38–40). However, the relationship between cysteine uptake and the pathogenesis of meningococcal infections currently remains unclear. The results obtained in the present study demonstrated that the uptake of cysteine by the meningococcal CTS was crucial for N. meningitidis survival/persistence in human endothelial cells.

## RESULTS

### Cysteine transport system (CTS) proteins, but not a sole Cbp protein, participated in meningococcal infections in HBMEC.

**Fig 1:**
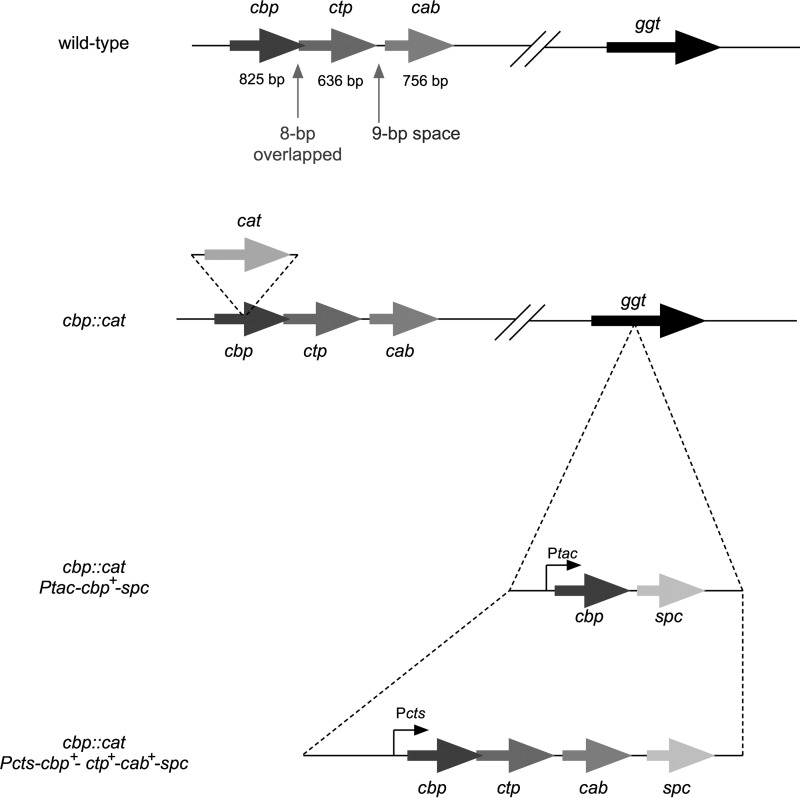
Schematic representation of wild-type strain and insertion mutant in the *cbp* gene and ectopic complementation of *Ptac cbp^+^* or *cbp^+^ ctp^+^ cab^+^* genes at the *ggt* locus in N. meningitidis strains.

The genes that participate in cysteine transport have already been assigned by genome sequencing and annotation analyses in N. meningitidis strain MC58 (41). In the present study, NMB0787 (amino acid ABC transporter substrate-binding protein), NMB0788 (amino acid ABC transporter permease), and NMB0789 (amino acid ABC transporter ATP-binding protein) were renamed cysteine-binding protein (*cbp*), cysteine transporter permease (*ctp*), and cysteine ATP-binding protein (*cab*), respectively (Fig. 1) and represent the cysteine transport system (CTS). In the present study, we constructed an insertion mutation in the *cbp* gene and analyzed its biological function in meningococcal nutrient virulence.

**Fig 2:**
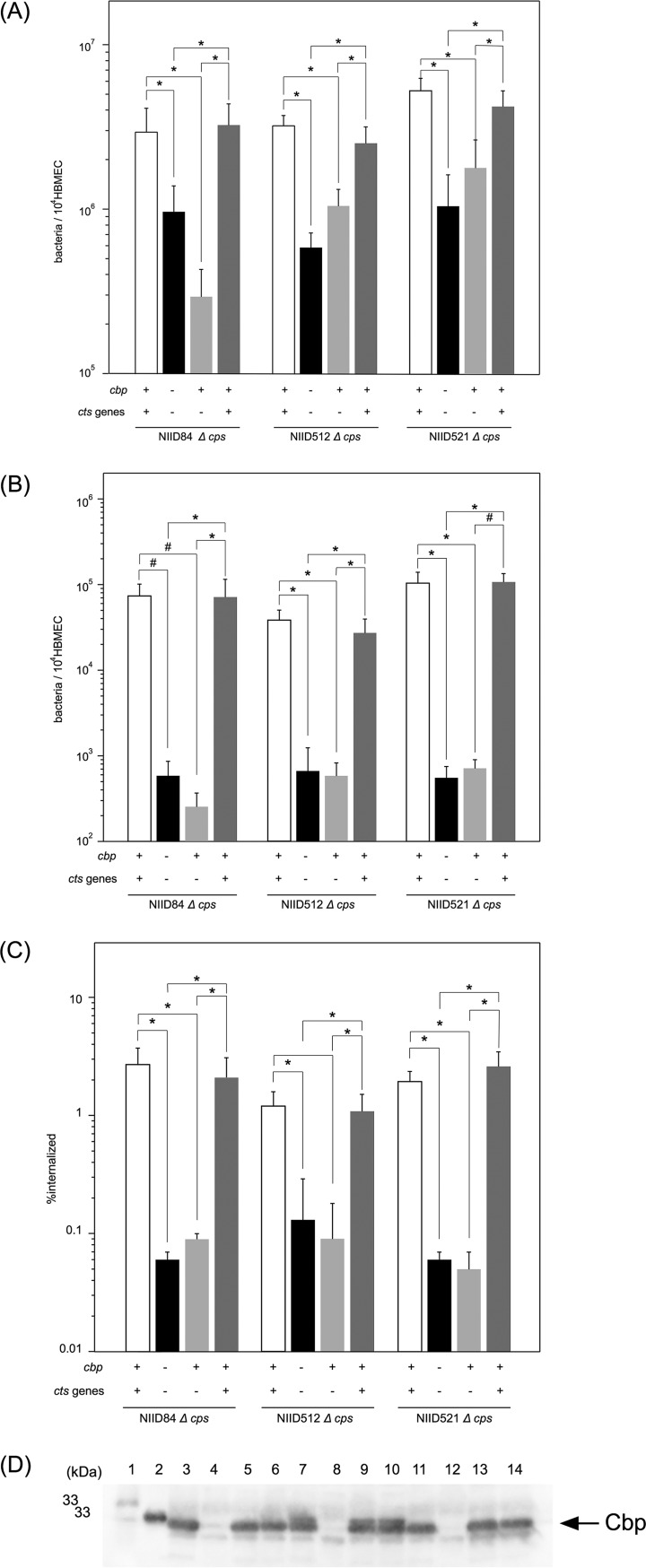
Mutation in *cts* genes affected apparent N. meningitidis internalization into HBMEC. (A to C) Adherence (A), internalization (B), and ratio of internalization/adhesion (percent internalized) (C) of *cbp* N. meningitidis mutants to HBMEC and effects of complementation of the *cbp^+^* or *cbp^+^ ctp^+^ cab^+^* genes in the *cbp* mutant on bacterial infection. The numbers of bacteria were measured as CFU. Levels of internalized bacteria were determined as numbers of bacteria recovered after the gentamicin treatment. Each value represents the mean ± standard error of mean (CFU per 10^4^ HBMEC) of results from at least four experiments. Open, filled, light gray, and dark gray bars in panels A, B, and C indicate the bacterial number of or percent internalized N. meningitidis *cts^+^* strains (NIID84 Δ*cps*, NIID512 Δ*cps*, and NIID521 Δ*cps*), *cbp*::*cat* (HT1959, HT2056, and HT2063), and Δ*cps* mutants in which the *cbp^+^* or *cts^+^* (*cbp^+^ ctp^+^ cab^+^*) genes were ectopically complemented (HT2077, HT2078, and HT2079 or HT2080, HT2081, and HT2082), respectively (see Table 1). *, *P < *0.001; #, *P < *0.02 (significantly different from the *cts^+^* strain or the Δ*cps* mutant complemented with *cts^+^* genes). (D) Western blotting for Cbp proteins. Bacterial extracts equivalent to an OD_600_ of 0.025 were analyzed by Western blotting. Lanes 1 and 2 represent molecular markers corresponding to 33 and 30 kDa, respectively. Lane 3, NIID84 Δ*cps* (*cts^+^*); lane 4, HT1959 (NIID84 Δ*cps cbp*); lane 5, HT2077 (NIID84 Δ*cps cbp ggt*::*cbp^+^*); lane 6, HT2080 (NIID84 Δ*cps cbp ggt*::*cts^+^*); lane 7, NIID512 Δ*cps* (*cts^+^*); lane 8, HT2056 (NIID512 Δ*cps cbp*); lane 9, HT2078 (NIID512 Δ*cps cbp ggt*::*cbp^+^*); lane 10, HT2081 (NIID512 Δ*cps cbp ggt*::*cts^+^*); lane 11, NIID521 Δ*cps* (*cts^+^*); lane 12, HT2063 (NIID521 Δ*cps cbp*); lane 13, HT2079 (NIID521 Δ*cps cbp ggt*::*cbp^+^*); lane 14, HT2082 (NIID521 Δ*cps cbp ggt*::*cts^+^*). The black arrow shows the Cbp protein in N. meningitidis.

Infectious abilities in human brain microvascular endothelial cells (HBMEC) were initially examined. The growth rate of the *cbp* mutant in assay medium (AM) (see Materials and Methods) was approximately one-third that of the wild-type strain in a 4-h incubation with shaking (data not shown). The adhesion ability of the *cbp* mutant HT1959 strain was reduced to approximately one-third that of the wild-type strain (Fig. 2A), which might reflect the growth rate difference in AM. On the other hand, the number of internalized bacteria decreased to approximately 1/100 that of wild-type strain NIID84 Δ*cps* (Opa^−^, Opc^+/−^) (Fig. 2B; see also Fig. S1 in the supplemental material). It was important that the same phenotype was observed in the other meningococcal strains, NIID512 (Opa^+^, Opc^−^) and NIID521 (Opa^+^, Opc^+/−^) (Fig. 2A and B; see also Fig. S1). In order to confirm the relationship between insertional mutation and infection defects, HT 2077, a *cbp* mutant complemented with the wild-type *cbp^+^* gene expressed from the *tac* promoter in *trans* at the *ggt* locus (Fig. 1) was constructed and examined in HBMEC. It is important that the insertion of genes at the *ggt* locus did not affect meningococcal infection of human endothelial and epithelial cells (32, 33, 38, 42, 43). While the Cbp protein was expressed in HT2077 (Fig. 2D), the infectious defect was not restored in HT2077 (Fig. 2B and C). In contrast, the infectious defect in HBMEC was restored by complementation with three wild-type *cts* genes (*cbp^+^*, *ctp^+^*, and *cab^+^*) in *trans* at the *ggt* locus in the HT2080 N. meningitidis strain (Fig. 1 and 2B and C). It is important that the same results were again obtained in the NIID512 and NID521 genetic backgrounds (Fig. 2). The transcription of *cts* genes in these mutants examined by reverse transcriptase PCR (RT-PCR) was consistent with the infection recovery in Fig. 2; *cbp* mRNA was detected in NIID84 Δ*cps*, HT2080, and HT2077, in which only the *cbp* gene was complemented, but *ctp* and *cab* mRNA were detected only in NIID84 Δ*cps* and HT2080, indicating that the *cbp*, *ctp*, and *cab* genes were transcriptionally comprised of one operon at the *cts* gene allele (Fig. S2). Taken together, these results suggested that all three *cts* genes, but not *cbp* alone, were required for the meningococcal infection of HBMEC. Moreover, considering the expression profiles of Opa and Opc (Fig. S1), which also affect meningococcal infection of cultured human cells (44–46), these proteins could not be related to the results observed in this study.

10.1128/mBio.02332-18.1 Fig S1 Expression of Opa (A) and Opc (B) proteins in N. meningitidis strains used in this study was examined by Western blotting. N. meningitidis, grown on GC agar plates, was suspended in PBS. Bacteria at an OD_600_ of 5 were harvested and resuspended in 1 ml 1× SDS buffer and boiled for 10 min, and 5-μl volumes of the samples were analyzed by 12% SDS-PAGE. Western blotting was performed as described previously (38). Lane 1, HT1132 (ST-44, Opa^−^/Opc^++^) (63); lane 2, HT1125 (ST-2032, Opa^−/+^/Opc^−^) (63); lane 3, NIID84 *cps*^−^; lane 4, NIID512 *cps*^−^; lane 5, NIID521 *cps*^−^. HT1132 and HT1125 strains were used for positive control of expression for Opc and Opa, respectively (63). Download FIG S1, TIF file, 0.4 MB. Copyright © 2018 Takahashi et al. 2018 Takahashi et al. This content is distributed under the terms of the Creative Commons Attribution 4.0 International license.

10.1128/mBio.02332-18.2 Fig S2 RT-PCR to examine the expression of *cts* genes as an operon. (A) Schematic representation of primer positions for RT-PCR. Three primer sets, including primer set cbp-RT-5 (GTGCCGATGCAACCCTGAACGAC) and cbp-RT-6 (CCACGGCTTCGTCATTGCCCTTGT), primer set ctp-RT-1 (GCGCCGATATGATTGTCAGCGCGT) and ctp-RT-2 (ACAGCGGCGTACCGCGAATGACGG), and primer set cab-RT-1 (GCAGGTGGTCGTCATCCTCGGGCC) and cab-RT-2 (CCTGTACGGCAACCGGTCCTTCCA), were used for amplification of 150-bp, 200-bp, and 250-bp DNA fragments from *ctp*, *ctp*, and *cab* mRNA, respectively, by RT-PCR. (B to D) RT-PCR was performed to detect mRNA of *cbp* (B), *ctp* (C), and *cab* (D) genes. Lanes 1 and 5, NIID84 Δ*cps* (*cts^+^*); lanes 2 and 6, HT1959 (NIID84 Δ*cps cbp*); lanes 3 and 7, HT2077 (NIID84 Δ*cps* Δ*cbp ggt*::*cbp^+^*); lanes 4 and 8, HT2080 (NIID84 Δ*cps cbp ggt*::*cts^+^*). Lanes 1, 2, 3, and 4 indicate the results without reverse transcriptase reaction (see Materials and Methods). The black arrows indicate DNA products amplified from *cbp* (B), *ctp* (C), and *cab* (D) mRNA, respectively. Download FIG S2, TIF file, 1.9 MB. Copyright © 2018 Takahashi et al. 2018 Takahashi et al. This content is distributed under the terms of the Creative Commons Attribution 4.0 International license.

Therefore, the subsequent experiments were performed using the NIID84 Δ*cps* strain only.

### Cysteine uptake activity via the CTS correlated with the meningococcal infection of HBMEC.

**Fig 3:**
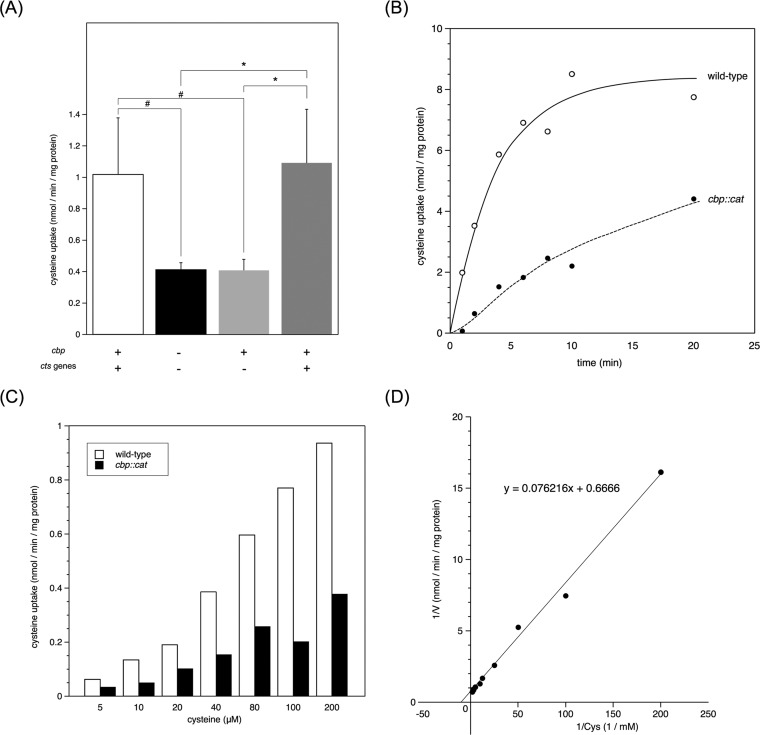
l-Cysteine uptake into N. meningitidis strains. (A) Effects of the *cbp* mutation and complementation of wild-type genes in N. meningitidis on l-cysteine uptake. The assay was performed as described in Materials and Methods in the presence of 100 μM l-[^35^S]cysteine and a final NaCl concentration of 60 mM in SM. Transport was examined for 5 min due to its lower efficiency than l-glutamate uptake (32). Open, filled, light gray, and dark gray bars indicate the cysteine uptake rate (nmol/min/mg protein) of NIID84 Δ*cps* (*cts^+^*), HT1959 (NIID84 Δ*cps* *cbp*), HT2077 (NIID84 Δ*cps* *cbp ggt*::*cbp^+^*), and HT2080 (NIID84 Δ*cps* *cbp ggt*::*cts^+^*), respectively. #, *P < *0.02 (significantly different from the *cts^+^* strain); *, *P < *0.005 (significantly different from the Δ*cps cts^+^* strain). (B) Time course of l-cysteine uptake. The assay was performed in the presence of 100 μM l-[^35^S]cysteine and a final NaCl concentration of 60 mM in SM. The open circles with a solid line and filled circles with a dashed line indicate cysteine uptake by NIID84 Δ*cps* (*cts^+^*) and HT1959 (NIID84 Δ*cps* *cbp*), respectively. (C) Concentration dependence of l-cysteine import by strains NIID84 Δ*cps* (*cts^+^*) and HT1959 (NIID84 Δ*cps* *cbp*). The assay was performed in SM containing 60 mM NaCl for 5 min. (D) Lineweaver-Burk plot of data shown in panel C relative to NIID84 Δ*cps* (*cts^+^*). A formula deduced from the data is also shown in the same panel. The *K_m_* value of meningococcal [^35^S]cysteine uptake in SM was calculated to be approximately 114 μM.

While all three proteins (Cbp, Ctp, and Cab) were clearly required for the efficient internalization of HBMEC, the relationship between the pathogen’s infectious abilities and cysteine transport via the CTS remained unknown. Therefore, we investigated cysteine uptake activity. In our previous studies, l-glutamate uptake activity via the GltT-GltM transport system was examined in buffer A containing 20 mM NaCl for 20 s (32) because l-glutamate uptake is drived by a Na^+^ gradient (31). However, under the same conditions, [^35^S]cysteine was imported very inefficiently into N. meningitidis (see Fig. 3A compared to Fig. S3A). Moreover, the addition of NaCl did not promote uptake (data not shown). Thus, in the present study, synthetic medium (SM) devoid of cysteine (see Materials and Methods) was used instead of buffer A containing NaCl. Since increases in NaCl concentrations in SM did not stimulate meningococcal cysteine uptake (Fig. S3C), meningococcal cysteine uptake was examined in SM containing 60 mM NaCl in the present study. Under these conditions, [^35^S]cysteine was imported less efficiently in *cbp* mutant HT1959 than in wild-type strain NIID84 Δ*cps* (Fig. 3A). The low efficiency of cysteine uptake was not restored in HT2077 (a *cbp* mutant complemented with a *cbp^+^* gene only) but recovered in the HT2080 N. meningitidis strain (a *cbp* mutant complemented with all three *cts^+^* genes) (Fig. 3A). The correlation observed with infectious abilities in HBMEC (Fig. 2B and C) suggested that the efficient influx of extracellular cysteine via the CTS into N. meningitidis contributed to the efficient meningococcal internalization into HBMEC (see Discussion).

10.1128/mBio.02332-18.3 Fig S3 (A) l-Cysteine uptake into N. meningitidis strains in buffer A containing 100 mM NaCl. Assay conditions were the same as those described for SM (see Materials and Methods), and results of transport performed for 5 min were examined. Open and filled bars indicate the cysteine uptake rates (in picomoles per minute per milligram of protein) of NIID84 Δ*cps* (*cts^+^*) and NIID84 Δ*cps* (*cbp*), respectively, while cysteine uptake by HT1959 was not observed under these conditions. (B) Time course of l-cysteine uptake. The assay was performed in the presence of 100 μM l-[^35^S]cysteine and NaCl at a final concentration of 100 mM in buffer A. The open circle with a solid line and the filled circle with a dashed line indicate cysteine uptake by NIID84 Δ*cps* (*cts^+^*) and HT1959 (NIID84 Δ*cps cbp*), respectively. (C) NaCl dependence of l-cysteine import by strains NIID84 Δ*cps* (*cts^+^*) and HT1959 (NIID84 Δ*cps cbp*) in SM. The assay was performed in SM containing the indicated concentration of NaCl in the presence of 100 μM l-[^35^S]cysteine for 5 min. Download FIG S3, TIF file, 0.7 MB. Copyright © 2018 Takahashi et al. 2018 Takahashi et al. This content is distributed under the terms of the Creative Commons Attribution 4.0 International license.

### Ezrin accumulation was observed beneath the *cbp* N. meningitidis mutant.

**Fig 4:**
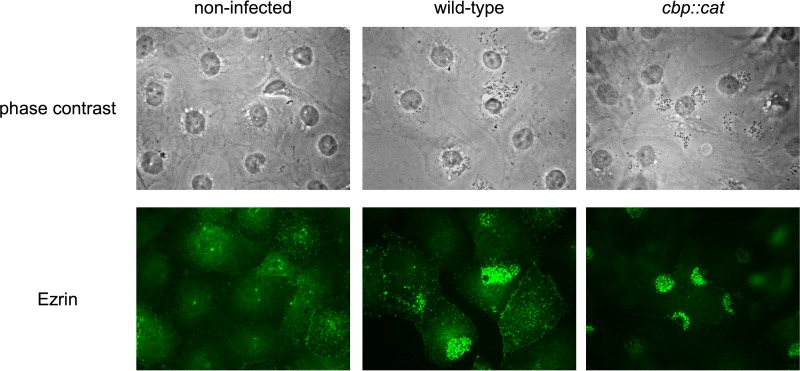
Immunofluorescence microscopy showing the accumulation of ezrin beneath N. meningitidis strains. The HBMEC monolayer was infected with wild-type *cts^+^* (middle) and *cbp*::*cat* (right) N. meningitidis strains. A noninfected HBMEC monolayer is also shown in the left panels. Bacteria and HBMEC were observed by phase-contrast microscopy (upper panels). Ezrin was immunostained with anti-ezrin monoclonal antibodies (MAb) and Alexa Fluor 488-conjugated rabbit anti-mouse IgG (green channel) (lower panels).

We previously demonstrated that the transient uptake of l-glutamate upon meningococcal adhesion to HMBEC triggered meningococcal internalization into HBMEC and the concomitant accumulation of ezrin beneath meningococci (33), which is a marker for the meningococcal stimulation of host cell signaling for internalization (47). In order to establish whether the same strategy is applicable to cysteine uptake, we analyzed changes in the host cell cytoskeleton upon meningococcal infection using indirect immunofluorescence to monitor the localization of ezrin (Fig. 4). While the accumulation of ezrin was minimal in HBMEC not infected with N. meningitidis (Fig. 4, left panels), it was clearly detected beneath wild-type N. meningitidis strain NIID84 Δ*cps* (Fig. 4, middle panels). It is important that ezrin also accumulated beneath the *cbp* HT1959 mutant as efficiently as beneath the wild-type strain (Fig. 4, right panels), while the ezrin accumulation was not observed in the *ΔgltT ΔgltM* N. meningitidis strain (32, 33). This result indicated that the *cbp* mutant was not defective for stimulation of the ezrin accumulation but was defective in another mechanism(s).

### The intracellular survival rate of the *cbp* mutant was reduced to the level seen with the *ΔgshB* mutant.

**FIG 5:**
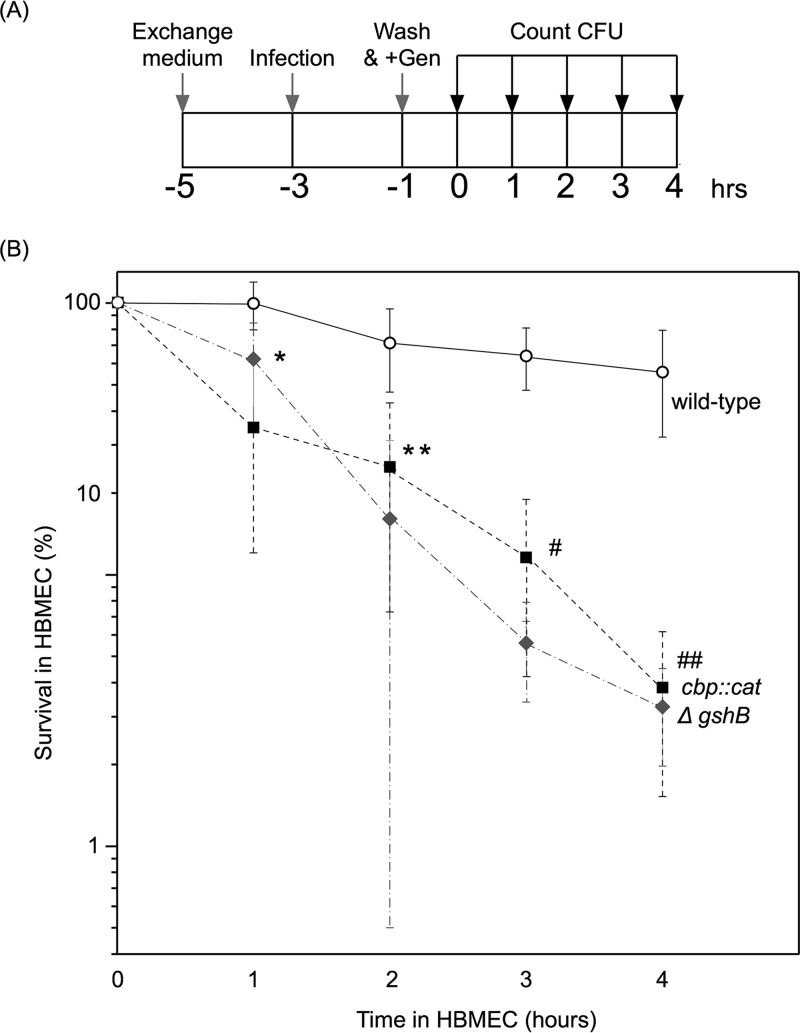
Percentage of survival of intracellular bacteria after killing of extracellular bacteria with gentamicin. (A) Diagram of the protocol to monitor the number of intracellular bacteria after a 2-h infection. Gen, gentamicin. Detailed procedures are described in Materials and Methods. (B) Percentage of survival of intracellular bacteria in HBMEC. Percentages of survival were calculated as 100 × (CFU at the indicated time/CFU at the removal of gentamicin). The open circle, filled square, and gray diamond shapes indicate the percentages of survival of *ctp^+^* (NIID84 Δ*cps*), *cbp*::*cat* (HT1959), and *ΔgshB*::*spc* (HT2120) N. meningitidis strains, respectively. Ranges represent mean percentages of survival in at least five experiments, and error bars represent the standard errors of the means. *, *P < *0.01; **, *P < *0.05; #, *P < *0.001; ##, *P < *0.002 (significantly different from the wild-type *cts^+^* strain).

Since defective l-glutamate uptake reduced glutathione synthesis and concomitantly decreased intracellular survival (31–34), it is possible that the invasion defect in the *cbp* mutant can be explained by bacterial survival/persistence in HBMEC after internalization. In order to examine this possibility, the meningococcal survival rate in HBMEC was assessed in a 4-h incubation under our experimental conditions (Fig. 5A). The intracellular bacterial number of wild-type N. meningitidis strain NIID84 Δ*cps* gradually decreased during the course of the incubation, and after a 4-h incubation, approximately 60% of the bacteria remained in HBMEC (Fig. 5B). In contrast, the intracellular bacterial number of the *cbp* N. meningitidis mutant markedly decreased during the 4-h incubation (Fig. 5B). The rate of the decrease (6% of the zero time number) in the *cbp* mutant was markedly less than that (60% of the zero time number) of the wild-type strain. These results suggest that the *cbp* mutation decreased intracellular survival rates in HBMEC, which may have contributed to the apparent decrease in the internalized bacterial number in HBMEC (Fig. 2B).

### Glutathione concentrations did not markedly decrease in the *cbp* N. meningitidis mutant.

Previous studies reported that l-glutamate uptake is important for the synthesis of glutathione, a ubiquitous antioxidant in all living cells (48), and leads to N. meningitidis resistance to the neutrophil oxidative burst (34) and increased intracellular survival in human cells (31, 33). Since cysteine is also one of the components of glutathione, we constructed the *gshB* (glutathione synthetase) HT2120 mutant and examined the relationship between glutathione synthesis and the apparent ability of the *cbp* N. meningitidis mutant to internalize into HBMEC (Fig. 2B). While meningococcal adhesion to HBMEC was slightly affected by the *cbp* and *gshB* mutations, a marked decrease was not observed (Fig. S4A). On the other hand, the internalization of the *cbp* HT1959 mutant into HBMEC was approximately 100-fold less efficient than that of wild-type strain NIID84 Δ*cps*, while that of the *gshB* HT2120 mutant was approximately 3-fold less efficient than that of wild-type strain NIID84 Δ*cps* (Fig. S4B and C). These results suggest that glutathione synthesis was less likely to be related to the apparent meningococcal internalization (intracellular survival) into HBMEC.

10.1128/mBio.02332-18.4 Fig S4 A deficiency in the production of glutathione did not markedly affect meningococcal internalization into HBMEC. Data represent (A) adherence and (B) internalization and (C) the ratio of internalization/adhesion (percent internalized) of *cbp* or *ΔgshB* N. meningitidis mutants to HBMEC. The numbers of bacteria were measured as CFU. Internalized bacteria were assessed as bacteria recovered after the gentamicin treatment. Values represent means ± standard errors of the means (CFU per 10^4^ HBMEC) of results from at least four experiments. Open, filled, and dark gray bars in panels A, B, and C indicate the bacterial numbers of the N. meningitidis *cts^+^* (NIID84 Δ*cps*) strain, the *cbp*::*cat* (HT1959) strain, and the *ΔgshB*::*spc* mutant (HT2120), respectively (see Table 1). *, *P < *0.005 (significantly different from the *cts^+^* or *ΔgshB* strain). Download FIG S4, TIF file, 0.4 MB. Copyright © 2018 Takahashi et al. 2018 Takahashi et al. This content is distributed under the terms of the Creative Commons Attribution 4.0 International license.

**FIG 6:**
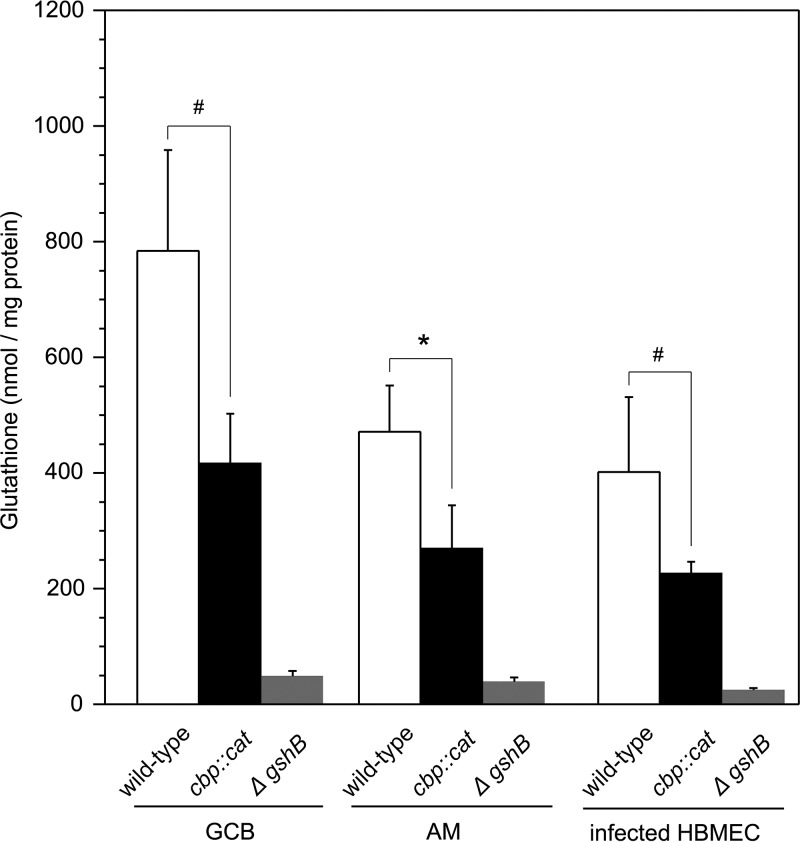
Intracellular glutathione concentrations in N. meningitidis. The intracellular glutathione concentration in N. meningitidis was measured as described in Materials and Methods. The glutathione concentration in bacteria is expressed as nanomoles of glutathione per mg of bacterial protein. Ranges represent the meand of intracellular glutathione concentrations in N. meningitidis in at least four experiments, and error bars represent the standard errors of the means. #, *P < *0.05; *, *P < *0.003 (significantly different from the wild-type *cts^+^* strain).

In order to further clarify whether glutathione participates in intracellular survival, the content of glutathione was measured (Fig. 6). In N. meningitidis strains grown in gonococcal (GC) medium base (GCB), the intracellular glutathione concentration in the *ΔgshB* mutant was approximately 50 μmol/mg protein, representing the background level of glutathione during the 4-h incubation (Fig. 6). Under the same conditions, wild-type N. meningitidis strain NIID84 Δ*cps* had an intracellular glutathione concentration of approximately 800 μmol/mg protein, while that in the *cbp* mutant was approximately 400 μmol/mg protein (Fig. 6). When the strains were grown in AM, intracellular glutathione concentrations slightly decreased in the wild-type N. meningitidis strain but remained unchanged in the *cbp* mutant. Similar results were observed for N. meningitidis strains infecting HBMEC (Fig. 6). Moreover, the reduction in the glutathione amount was statistically recovered in a *cbp* mutant complemented with HT2080 *cts* genes but not in a *cbp* mutant complemented with a HT2077 *cbp* gene (Fig. S5A). These results suggest that cysteine uptake via the CTS accounts for some, but not all, of the glutathione synthesis.

10.1128/mBio.02332-18.5 Fig S5 Genetic complementation of *cts* genes, but not of a *cbp* gene, in a *cbp* mutant recovered the reduction of glutathione (A) and resistance to H_2_O_2_ (B) and paraquat (C). Open filled dark gray bars and light gray bars in panels A, B, and C indicate intracellular glutathione concentrations (A), sensitivity of N. meningitidis strains to H_2_O_2_ (B), and sensitivity of N. meningitidis strains to paraquat (C) for N. meningitidis strains NIID84 Δ*cps* (*cts^+^*), HT1959 (NIID84 Δ*cps cbp*), HT2077 (NIID84 Δ*cps cbp* *ggt*::*cbp^+^*), and HT2080 (NIID84 Δ*cps cbp ggt*::cts+) (see Table 1). Ranges represent the mean glutathione concentrations or percentages of survival measured in at least three experiments, and error bars show standard errors of the means. * (*P < *0.05) and # (*P = *0.07), significantly different from the results determined for strains NIID84 Δ*cps* and HT2080, respectively. Download FIG S5, TIF file, 0.7 MB. Copyright © 2018 Takahashi et al. 2018 Takahashi et al. This content is distributed under the terms of the Creative Commons Attribution 4.0 International license.

### Resistance to reactive oxygen species (ROS) was not largely defective in the *cbp* N. meningitidis mutant.

**FIG 7:**
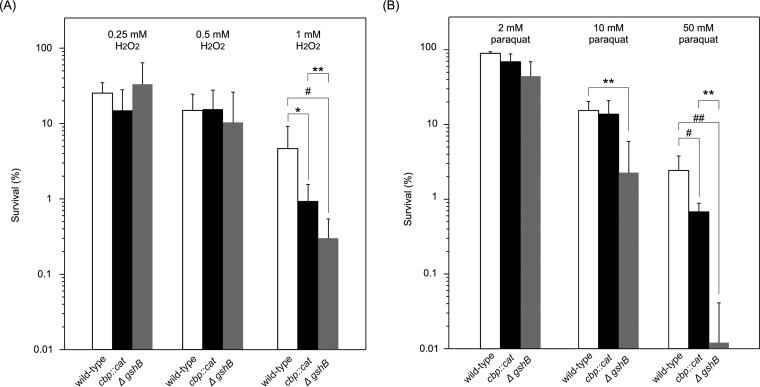
Sensitivity of N. meningitidis strains to H_2_O_2_ and paraquat. Bacteria were incubated at 37°C in GCB with the indicated concentration of H_2_O_2_ for 15 min (A) and paraquat for 60 min (B). The number of surviving bacteria was counted as CFU by plating onto GC agar plates with appropriate dilutions. Ranges represent the mean percentages of survival in at least five experiments, and error bars show the standard errors of the means. *, *P < *0.05; #, *P < *0.02; **, *P < *0.001; ##, *P < *0.003 (significantly different from the wild-type *cts^+^* strain or *ΔgshB*::*spc* mutant).

l-Glutamate imported via GltT-GltM transporters was previously shown to be utilized in the synthesis of glutathione for resistance to H_2_O_2_ (34), a membrane-permeable ROS that damages proteins and DNA (49). Since glutathione concentrations were reduced to approximately 50% of the level in the wild-type strain (Fig. 6), decreases in the glutathione concentration in the *cbp* mutant may have reduced the intracellular survival rate in HBMEC. In order to examine this possibility, sensitivity to H_2_O_2_ and paraquat, which also generates toxic oxygen species during respiration within the cytoplasm after penetrating the cells (50, 51), was investigated *in vitro* (Fig. 7). The levels of sensitivity to 0.25 and 0.5 mM H_2_O_2_ did not significantly differ among the wild-type, *cbp* mutant, and *ΔgshB* N. meningitidis strains (Fig. 7A). However, at 1 mM H_2_O_2_, the *cbp* mutant was approximately 5-fold more sensitive than the wild-type N. meningitidis strain under conditions in which the *ΔgshB* mutant was approximately 13-fold more sensitive than the wild-type N. meningitidis strain (Fig. 7A). Under these conditions, the *cbp* mutant was approximately 3-fold less sensitive to 1 mM H_2_O_2_ than the *ΔgshB* mutant (*P < *0.001). These results suggest that a cysteine transport deficiency resulted in N. meningitidis being slightly more sensitive to 1 mM H_2_O_2_. The slight reduction of resistance to 1 mM H_2_O_2_ was statistically recovered in a *cbp* mutant complemented with HT2080 *cts* genes but not in a *cbp* mutant complemented with a HT2077 *cbp* gene (Fig. S5B). Regarding paraquat, no significant differences were observed with 2 mM paraquat among the wild-type, *cbp* mutant, and *ΔgshB* N. meningitidis strains (Fig. 7B). At 10 mM paraquat, no significant differences were noted between the wild-type and *cbp* mutant N. meningitidis strains, while the *ΔgshB* mutant was approximately 7-fold more sensitive than the wild-type and *cbp* mutant N. meningitidis strains (Fig. 7B). However, at 50 mM paraquat, the *cbp* mutant was approximately 3-fold more sensitive than the wild-type strain whereas the *ΔgshB* mutant was approximately 240-fold more sensitive than the wild-type strain (Fig. 7B). The reduction of resistance to 50 mM paraquat was statistically recovered in a *cbp* mutant complemented with HT2080 *cts* genes but not in a *cbp* mutant complemented with a HT2077 *cbp* gene (Fig. S5C). Collectively, these results with respect to sensitivities to H_2_O_2_ and paraquat indicated that decreases in glutathione concentrations due to CTS defects did not markedly affect meningococcal resistance to ROS.

### Cysteine uptake via the CTS was essential for meningococcal growth in the presence of less than 300 μM cysteine.

**FIG 8:**
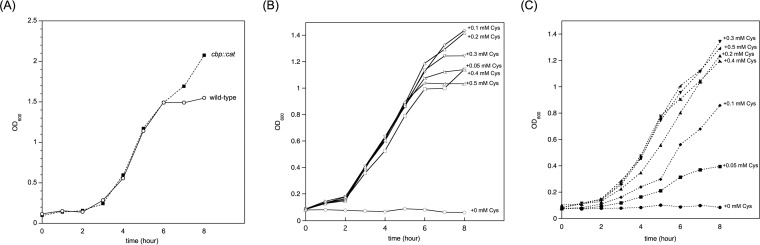
Growth of N. meningitidis strains in liquid media. Meningococcal growth was monitored at OD_600_. (A) Meningococcal growth in GCB. Open circles with a solid line and filled circles with a dotted line correspond to wild-type N. meningitidis strain NIID84 Δ*cps* and *cbp*::*cat* mutant HT1959, respectively. Figures show representative data from at least three experiments with similar results. (B and C) Growth of wild-type N. meningitidis strain NIID84 Δ*cps* (B) and *cbp*::*cat* N. meningitidis mutant HT1959 (C) in SM devoid of cysteine supplemented with the indicated concentration of cysteine. The following marks indicate the concentration of cysteine supplemented in SM: circle, 0 mM (not supplemented); square, 0.05 mM; diamond, 0.1 mM; triangle, 0.2 mM; inverted triangle, 0.3 mM; right triangle with a right angle on the left, 0.4 mM; right triangle with a right angle on the right, 0.5 mM. Solid lines with open marks and dotted lines with filled marks indicate the growth of wild-type and *cbp*::*cat* N. meningitidis strains, respectively.

We investigated meningococcal growth in liquid media. In the complete medium GCB, the *cbp* mutant grew as efficiently as the wild-type N. meningitidis strain (Fig. 8A), indicating that the growth of the *cbp* mutant was not defective in liquid medium. In previous experiments using meningococcal defined medium, Neisseria chemically defined medium (NCDM), developed by Catlin (30), was commonly used for N. meningitidis (31, 34). However, under our experimental conditions, most of the N. meningitidis strains did not grow in NCDM or grew to an optical density at 600 nm (OD_600_) of ∼0.4 from 0.1 at the starting point even though N. meningitidis had been precultured on GC agar (data not shown) (38). In order to overcome this issue, we used our original SM, which was basically the same as NCDM but was supplemented with a one-fourth volume of originally reconstituted MCDB131 devoid of NaCl and cysteine (see Materials and Methods and Table S2 in the supplemental material). Thus, SM did not contain NaCl or cysteine, and these reagents were supplemented if indicated. In SM supplemented with 60 mM NaCl, wild-type N. meningitidis strain NIID84 Δ*cps* did not grow when cysteine was not supplemented (Fig. 8B). On the other hand, supplementation of cysteine completely suppressed the growth defect (Fig. 8B), ensuring that meningococcal growth in SM was completely dependent on cysteine. It is important that the supplementation of even 50 μM cysteine was sufficient for meningococcal growth in SM (Fig. 8B). Regarding the growth of the *cbp* mutant, it also did not grow in SM devoid of cysteine (Fig. 8C). The supplementation of increasing concentrations of cysteine gradually suppressed the growth defect in the *cbp* mutant, while at least 300 μM cysteine was required in order to completely suppress the growth defect (Fig. 8C). These results suggest that cysteine uptake via the CTS is crucial for meningococcal growth under conditions of restricted concentrations of cysteine. Furthermore, these results are consistent with the *K_m_* value of meningococcal [^35^S]cysteine uptake in SM being approximately 114 μM (Fig. 3D). Since the concentration of the intracellular cysteine pool is very limited (approximately 100 μM) (52), these results suggest that the observed marked reduction in bacterial internalization into HBMEC was mostly due to the meningococcal starvation of cysteine in HBMEC (see Discussion).

## DISCUSSION

Investigation of the role of metabolism in virulence was recently recognized as a priority equivalent to studying classical virulence factors (24, 26, 53). While cysteine levels in eukaryotes need to be tightly regulated by controlling the degradation of this amino acid (54, 55) and transport of it from the extracellular milieu into the cell and vice versa (56), bacterial cysteine uptake has not yet been elucidated in detail (57–60). N. meningitidis cysteine transport and its role in the pathogenesis of meningococcal infections have yet to be clarified. To the best of our knowledge, while all of the experiments in this study were done only by the use of *in vitro* models and with a limited number of meningococcal strains, this is the first study to have suggested a relationship between meningococcal cysteine uptake and the pathogenesis of meningococcal infections.

Although the N. meningitidis strains used in the present study were cysteine auxotrophs (Fig. 8), a genome-scale metabolic model suggested that N. meningitidis is capable of the *de novo* synthesis of all amino acids, including cysteine (40, 61). However, experimental data revealed contrasting results; some N. meningitidis strains, namely, MC58 (62) and HB-1 (40), grew in minimal defined media, while strain α522 failed to grow (62). Previous studies also demonstrated that cysteine auxotrophs differed among N. meningitidis strains (30) and that many N. meningitidis strains had cysteine auxotroph characteristics with respect to their growth in the presence of low cysteine concentrations (38–40). While SM used in the present study contained approximately 3 mM inorganic sulfur molecules, which were mainly SO_4_^2−^ (see Table S2 in the supplemental material), NIID84 Δ*cps* did not grow in SM if cysteine was supplemented (Fig. 8B). Although further experiments are required to obtain more-comprehensive information, the following may be considered: N. meningitidis potentially has a set of genes for *de novo* cysteine synthesis in nature, and no mutation was found in the open reading frame (ORF) of any CTS genes in the genomes of 14 N. meningitidis strains recorded in NCBI database (data not shown). However, their products might not be always expressed in all N. meningitidis strains because of some negative on the transcription or translation, or enzymes other than CTS enzymes might affect cysteine assimilation but not acquisition for N. meningitidis. In fact, the levels of cysteine acquisition ability were different between meningococcal strains and seemed not to be related to the sequence type (see Fig. S6 in the supplemental material) or to the invasive/noninvasive phenotype (63). Regardless of the nature of the cysteine auxotroph in N. meningitidis, the meningococcal ability to uptake cysteine from the surrounding environment would be advantageous for N. meningitidis for the following reasons: N. meningitidis preferably uses cysteine (cystine) as a sulfur source (41), and *de novo* cysteine synthesis from inorganic sulfur molecules is more laborious and energetically less efficient than cysteine uptake from the surrounding environment because cysteine synthesis requires multiple enzymatic steps (41) with at least one ATP molecule (61). Furthermore, nonessential sulfur is not stored by N. meningitidis (36). Therefore, N. meningitidis may have to continuously and quickly acquire cysteine for its survival, persistence, and proliferation in humans. Additional experiments are required for more complete understanding of the meningococcal cysteine acquisition.

10.1128/mBio.02332-18.6 Fig S6 Growth of N. meningitidis sequence type (ST) variant strains in SM supplemented with 100 μM cysteine. Meningococcal growth was monitored at OD_600_. The following symbols indicate the ST strain variants: open circle, NIID84 Δ*cps* (ST-33); open square, HT1125 (ST-2032); filled circle, HT1336 (ST-5); filled square, HT1142 (ST-23); diamond, HT1143 (ST-198); right triangle with a right angle on the left, HT1034 (ST-32); triangle, HT1150 (ST-2046); right triangle with a right angle on the right, HT1129 (ST-11); inverted triangle, HT1132 (ST-44). The levels of expression of PilC, Opa, Opc, and PilE proteins and the ability to infect human cultured endothelial and epithelial cells of these *N.* meningitidis strains have been characterized previously (63). Download FIG S6, TIF file, 0.4 MB. Copyright © 2018 Takahashi et al. 2018 Takahashi et al. This content is distributed under the terms of the Creative Commons Attribution 4.0 International license.

Three pathogenic bacteria with auxotroph characteristics for cysteine have evolved their own strategies to acquire cysteine in host cells; intracellular Legionella pneumophila acquires nutrients that include cysteine by promoting host proteasomal degradation (25) and Anaplasma phagocytophilum obtains cysteine by promoting host autophagy (64). Francisella tularensis uses glutathione as a cysteine source for intracellular multiplication by γ-glutamyl aminopeptidase (GGT) (52), which is the same strategy used for meningococcal proliferation in CSF (38). However, the results obtained so far have indicated that the intracellular persistence of N. meningitidis was not supported by meningococcal GGT because N. meningitidis strain HT2022, in which the *ggt* gene was disrupted by the insertion of all three wild-type *cts* genes (Fig. 1), was internalized as efficiently as the wild-type strain (Fig. 2B and C). Regarding pathogenic bacteria that cause meningitis, such as Haemophilus influenzae and Streptococcus pneumoniae, while limited information is currently available on the role of cysteine in the pathogenesis of meningitis, the cysteine synthesis gene in S. pneumoniae was shown to be transcriptionally upregulated in the presence of low cysteine concentrations (65). While meningococcal glutamate uptake was also regulated by Na concentration (31), the meningococcal cysteine uptake activity was not regulated by Na (Fig. S3C), leading to the speculation that the activity might be not altered by environmental conditions.

Compared to the large reduction in the intracellular survival rate (Fig. 5B), [^35^S]Cys uptake in the *cbp* mutant decreased to only approximately 40% of the level seen in the wild-type strain (Fig. 3A). The discrepancy might have been due to the experimental conditions used for analysis of [^35^S]Cys uptake in this study; the [^35^S]Cys obtained in our laboratory tended to bind nonspecifically to meningococci as well as to any chemical filter (e.g., polyvinylidene difluoride [PVDF] and glass) even though they were washed with excess amounts of phosphate-buffered saline (PBS) containing 2% saponin (data not shown). Since no other cysteine uptake system was found in the meningococcal genome database uploaded (data not shown), the CTS would seem to be unique with respect to meningococcal cysteine acquisition, and it was also shown that the [^35^S]Cys uptake in the *cbp* mutant could not be observed even for 5 min of incubation with a high concentration of [^35^S]Cys in buffer A instead of SM (Fig. S3A). Thus, it would also seem to be possible that the [^35^S]Cys uptake examined in SM in this study was overestimated against the true function of cysteine uptake via the CTS in N. meningitidis and that methods used to address the problems regarding [^35^S]Cys uptake could be further improved.

**FIG 9:**
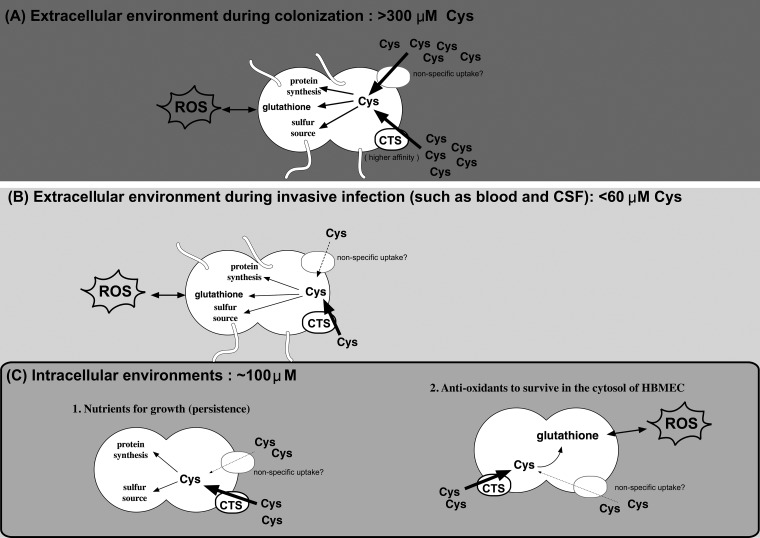
Schematic representation of meningococcal infections in humans. Since *cbp* N. meningitidis grew in cysteine-rich medium (GCB) (Fig. 8A), N. meningitidis may have two cysteine transport systems: a CTS that functions under low-cysteine conditions (higher affinity for cysteine) and an unidentified cysteine transporter that may function only under cysteine-rich conditions (lower affinity for cysteine). While this study showed only the intracellular functions of cysteine uptake via the CTS in N. meningitidis (C), this physiological function may be extensively applied to all human milieus for meningococcal infections in humans on the basis of the following concept. During the course of meningococcal invasion, until the point at which N. meningitidis reaches the human cells, N. meningitidis encounters at least 3 types of environments in humans: (A) the environment outside the human (e.g., nasopharynx), (B) the environment inside humans but not the intracellular milieu (e.g., blood and CSF), and (C) the intracellular milieu (in epithelial and endothelial cells). Cysteine is not limited in the environment represented in panel A because sufficient cysteine is supplied from the digestion of food. However, cysteine availability is very limited inside human environments (B and C) due to nutrient immunity with respect to cysteine. Cysteine concentrations in extracellular environments in humans (B) in blood (∼50 μM) and CSF (∼1 μM) are lower than in the intracellular milieu (A). The limitation of cysteine availability is also found in the intracellular environment, in which the concentration of cysteine is estimated to be approximately 100 μM. Thus, N. meningitidis has to “dive” into humans to cause IMD, in which context the milieu cysteine availability is always limited by the human nutrient immunity.

The *cbp* mutant grew normally (similarly to the wild-type strain) in GCB (Fig. 8A), eliminating the possibility that a mutation in meningococcal CTS itself affected essential N. meningitidis growth. It should be noted that the *cbp* mutant grew better than the wild-type strain in GCB at the stationary phase (Fig. 8A), which might be related to the negative effect of cysteine for bacterial growth (discussed below). On the other hand, under cysteine-limited conditions, while the supplementation of at least 50 μM cysteine was sufficient to suppress the growth defect in cysteine-free SM in the wild-type strain (Fig. 8B), supplementation of more than 300 μM cysteine was required for the complete suppression of growth of the *cbp* mutant (Fig. 8C). These results suggest that meningococcal cysteine uptake mediated via the CTS plays an essential role in N. meningitidis growth under conditions of low cysteine concentrations. On the basis of the cysteine concentrations seen in humans, meningococcal cysteine uptake via the CTS is advantageous for N. meningitidis (Fig. 9); the concentration of cysteine in the human nasopharynx remains unclear, but the nasopharynx is considered to be one of the nutritionally richest niches in humans because cysteine is supplied from the digestion of food. Alternatively, cysteine may be supplied from nasopharyngeal commensal bacteria. On the other hand, when N. meningitidis enters and passes through human epithelial and endothelial cells by endocytosis, the intracellular cysteine concentration is very low at approximately 100 μM (52). When the organism enters the bloodstream, the concentration of cysteine in human blood plasma is only approximately 30 to 60 μM (65, 66). During inflammation with sepsis, the cysteine concentration is significantly decreased (67, 68). N. meningitidis attaches again to endothelial cells comprising the BBB, passes through cells or loosens tight junctions (47), and enters the CSF. The concentration of cysteine in the CSF is very limited at less than 1 μM (38). Thus, cysteine concentrations are very low in humans (69) because mammals synthesize cysteine from methionine and serine, and cysteine may also be supplied from glutathione (70), which is an abundant and ubiquitous molecule in all organs and cell types (48). The limitation of the availability of “free” cysteine in humans may be regarded as a type of nutrient immunity. Therefore, the meningococcal cysteine acquisition via the CTS may play an important role as a “nutrient virulence factor” for N. meningitidis infections in humans with nutrient immunity for cysteine (Fig. 9).

In the present study, we used unencapsulated N. meningitidis strains for efficient internalization by eliminating the multiple effects of the capsule on meningococcal infections in humans. As discussed in our previous study (33), the meningococcal capsule may affect the intracellular survival ability (Fig. 5). However, since the majority of internalized bacteria are considered to remain unencapsulated until a phase variation occurs (8, 71), the intracellular survival of the unencapsulated state may also be important in the N. meningitidis infectious cycle. Furthermore, since the capsule expression does not hinder the import of amino acid into N. meningitidis (34), cysteine uptake in N. meningitidis is physiologically advantageous for the infection of humans regardless of the capsule expression state.

While cysteine is an important amino acid because it contains sulfur, which plays a vital role in the catalytic sites of many enzymes and participates in ion and redox metabolism (72), excessive amounts of cysteine are also harmful for many bacteria (73). For example, Escherichia coli, which is a prototroph for cysteine, did not synthesize excess amounts of (74) or store (75) cysteine by feedback pathways. This may also be the case for N. meningitidis because the supplementation of a higher concentration of cysteine appeared to inhibit meningococcal growth in SM at the late logarithmic phase (Fig. 8B) and the addition of 1 mM cysteine also appeared to inhibit meningococcal growth in SM (data not shown). Since cysteine auxotrophic organisms have to control intracellular cysteine concentrations at its uptake only, it seems reasonable to hypothesize that slow cysteine uptake via the CTS in N. meningitidis is among its advantageous biological functions.

The exploitation of host nutrients by pathogens is a major factor in host-pathogen interactions and is one of the fundamental aspects of infectious diseases; however, our knowledge of microbial metabolism in hosts is limited. Further studies on the mechanisms underlying microbial (including meningococcal) “nutritional virulence” and metabolism *in vivo* will provide a more comprehensive understanding of the central aspect of infectious diseases and result in the development of treatments and prevention strategies, such as the development of inhibitors for folate metabolism in pathogenic bacteria (76) or for iron acquisition using antibodies against N. meningitidis transferrin-binding proteins (77).

## MATERIALS AND METHODS

### Bacterial growth conditions.

N. meningitidis strains (stored at –80°C) were routinely grown on GC agar plates at 37°C in a 5% CO_2_ atmosphere (78). Brain heart infusion (Becton, Dickinson, USA) agar containing 3% defibrinated horse blood (Nihon Biotest, Japan) was used in the selection of kanamycin-resistant N. meningitidis strains (63). E. coli was grown on L plates or in L broth liquid cultures at 37°C. When required, antibiotics were added at the following concentrations: kanamycin at 150 μg/ml, chloramphenicol at 5 μg/ml, and spectinomycin at 75 μg/ml for N. meningitidis and kanamycin at 50 μg/ml and ampicillin at 50 μg/ml for E. coli. The N. meningitidis strains used in the present study are listed in Table 1.

**TABLE 1:** Strains used in this study

Strain	Genotype	Parent strain	Source
N. meningitidis strains			
NIID84	Wild-type strain (ST-33, serogroup B)		This study
NIID512	Wild-type strain (ST-687, serogroup B)		This study
NIID521	Wild-type strain (ST-8959, serogroup B)		This study
NIID84 Δ*cps*	*ΔsiaB ΔsiaD*::*kan*	NIID84	This study
HT1959	*ΔsiaB ΔsiaD*::*kan cbp*::*cat*	NIID84 Δ*cps*	This study
HT2077	*ΔsiaB ΔsiaD*::*kan cbp*::*cat ggt*::*Ptac cbp^+^ spc*	HT1959	This study
HT2080	*ΔsiaB ΔsiaD*::*kan cbp*::*cat ggt*::*cbp^+^ ctp^+^ cab^+^ spc*	HT1959	This study
NIID512 *cps*-	*ΔsiaB ΔsiaD*::*kan*	NIID512	This study
HT2056	*ΔsiaB ΔsiaD*::*kan cbp*::*cat*	NIID512 Δ*cps*	This study
HT2078	*ΔsiaB ΔsiaD*::*kan cbp*::*cat ggt*::*Ptac cbp^+^ spc*	HT2056	This study
HT2081	*ΔsiaB ΔsiaD*::*kan cbp*::*cat ggt*::*cbp^+^ ctp^+^ cab^+^ spc*	HT2056	This study
NIID521 Δ*cps*	*ΔsiaB ΔsiaD*::*kan*	NIID521	This study
HT2063	*ΔsiaB ΔsiaD*::*kan cbp*::*cat*	NIID521 Δ*cps*	This study
HT2079	*ΔsiaB ΔsiaD*::*kan cbp*::*cat ggt*::*Ptac cbp^+^ spc*	HT2063	This study
HT2082	*ΔsiaB ΔsiaD*::*kan cbp*::*cat ggt*::*cbp^+^ ctp^+^ cab^+^ spc*	HT2063	This study
HT2120	*ΔsiaB ΔsiaD*::*kan ΔgshB*::*spc*	NIID84 Δ*cps*	This study

Escherichia coli strains			
JM109	*endA1 gyrA96 hsdR17*(r_K_^−^ m_K_^+^) *mcrB^+^ recA1 relA1 supE44 thi-1 Δ*(*lac-proAB*) *F'*[*traD36*, *proAB*, *lacI*^q^*Z*ΔM15]		Nippon Gene
BL21(DE3)	*fhuA2* [*lon*] *ompT gal* (*λ DE3*) [*dcm*] *ΔhsdS λ DE3 = λ sBamHIo ΔEcoRI-B int*::(*lacI*::*PlacUV5*::*T7 gene1*) *i21 Δnin5*		NEB

GC broth (GCB) contained the following (per liter); proteose peptone, 15 g; NaCl, 5 g; soluble starch, 0.5 g; K_2_HPO_4_, 1 g; KH_2_PO_4_, 4 g; IsoVitaleX enrichment (Difco), 10 ml; 1 M NaHCO_3_, 5 ml; 1 M MgCl_2_, 10 ml.

SM in the present study basically consisted of NCDM (30), except that NaCl and cysteine were omitted (details were shown in Table S2 in the supplemental material). NaCl and cysteine were added when required. Additionally, due to another nutrient requirement(s), most meningococcal strains in our laboratory did not grow in NCDM (data not shown). In order to overcome this issue, MCDB131 (Thermo Fisher) lacking cysteine and NaCl, which was specifically manufactured by Cell Science & Technology Inst. Inc. (Japan), was added to a one-fourth volume in a final volume of 10 ml NCDM (details of the contents are shown in Table S2).

### Construction of meningococcal mutants.

N. meningitidis strains that did not produce a capsule were constructed by transformation with the purified chromosomal DNA of HT1034 (*ΔsiaB ΔsiaD*::*kan*) (63), and kanamycin-resistant clones were selected (Table 1). The transformation of N. meningitidis strains was performed as described previously (78).

In order to construct the N. meningitidis *cbp* insertion mutant, a 0.8-kb DNA fragment from N. meningitidis H44/76 chromosomal DNA containing the *cbp* gene was amplified with primers cbp-1 and cbp-2 (Table S1) by the use of PrimeSTAR Max DNA polymerase (TaKaRa Bio, Japan) and was cloned into the SmaI site of the pMW119 vector (Nippon Gene, Japan) (4.2 kb) to construct pHT1319 (5 kb). The 5-kb DNA region of pHT1319 was amplified with primers cbp-3 and cbp-4, which separated the *cbp* structural gene into 400 and 425 bp, respectively, by the use of PrimeSTAR Max DNA polymerase and was ligated with a chloramphenicol resistance gene (*cat*) to construct pHT1328. A 1.8-kb DNA fragment containing the *cbp* structural gene insertionally disrupted with the *cat* gene was amplified with primers cbp-1 and cbp-2 from pHT1328 and transformed into N. meningitidis strains, and chloramphenicol-resistant clones were selected, resulting in *cbp* insertional mutants (Table 1).

10.1128/mBio.02332-18.7 TABLE S1 Oligonucleotides used in this study. Download Table S1, DOCX file, 0.1 MB. Copyright © 2018 Takahashi et al. 2018 Takahashi et al. This content is distributed under the terms of the Creative Commons Attribution 4.0 International license.

A *cbp* mutant complemented with the *cbp^+^* gene at the *ggt* allele was constructed as follows. A 0.8-kb *cbp* gene was amplified with primers pTTQ-1-cbp-5 and M13-47-cbp-6. A 4.3-kb DNA fragment was also amplified from pTTQ18 (79) with primers pTTQ-2(PO+RBS) and M13-47 reverse (Table S1). These two DNA fragments were connected by the use of SLiCE (80). The resultant plasmid was named pHT1381. In order to insert a spectinomycin resistance gene (*spc*) downstream of the *cbp* gene, a 5.1-kb DNA fragment was amplified from pHT1381 with primers pTTQ-5 and pTTQ-6 (Table S1). A 1-kb *spc* gene was also amplified from pHT154 (81) with primers pTTQ-5′(15mer)-M13-RV-Long and pTTQ-6′(15mer)-M13-47-Long. These two DNA fragments were connected by the use of SLiCE, resulting in a plasmid that was named pHT1386. A 3-kb DNA fragment containing the *Ptac*, *cbp*, and *spc* genes was amplified from pHT1386 with primers ggt-5′(15mer)-ptac-13 and ggt-3′(15mer)-ptac-14 and was inserted into a BstXI site (at the middle of the *ggt* coding region) of pHT624, in which the *ggt* gene harbored by pHT195 (81) was subcloned into pMW119, resulting in pHT1387. A 4.5-kb DNA fragment containing the *ggt*::*Ptac cbp spc* genes was amplified with primers ggt-1 and ggt-2 (82) and transformed into the N. meningitidis *cbp*::*cat* mutants, and spectinomycin-resistant (Spc^r^) clones were selected, resulting in *cbp* insertional mutants ectopically complemented with the *cbp* gene expressed from the *tac* promoter at the *ggt* allele (Table 1).

A *cbp* mutant complemented with all three *cts* genes (*cbp*, *ctp,* and *cab*) at the *ggt* allele was constructed as follows: a 3.3-kb DNA fragment containing *cts* genes was amplified from NIID84 chromosomal DNA with primers cts operon-1 and cts operon-2 and cloned into the SmaI site of pMW119, resulting in pHT1391. In order to insert the *spc* gene downstream of the *cab* gene, a 7.6-kb DNA fragment was amplified from pHT1391 with primers M13-47′(15mer)-pMW(MCS)-down-1 and M13-RV′(15mer)-pMW(MCS)-down-2 (Table S1). A 1-kb *spc* gene was amplified from pHT154 with the universal primers M13-RV and M13-47, and these two DNA fragments were connected by the use of SLiCE, resulting in pHT1393. A 4.3-kb DNA fragment containing *cts* genes and the *spc* gene was amplified from pHT1393 with primers ggt-5′(15mer)-pMW119-F and ggt-3′(15mer)-pMW119-R (Table S1) and cloned into a BstXI site of pHT624 by the use of SLiCE, resulting in a plasmid that was named pHT1395. A 6.3-kb DNA fragment containing the *ggt*::*cbp ctp cab spc* genes was amplified from pHT1395 with primers ggt-1 and ggt-2 and transformed into the N. meningitidis *cbp*::*cat* mutants, and Spc^r^ clones were selected, resulting in *cbp* insertional mutants ectopically complemented with the *cts* genes expressed from their own promoter at the *ggt* allele (Table 1).

10.1128/mBio.02332-18.8 TABLE S2 Concentrations of synthetic medium used in this study. Download Table S2, DOCX file, 0.1 MB. Copyright © 2018 Takahashi et al. 2018 Takahashi et al. This content is distributed under the terms of the Creative Commons Attribution 4.0 International license.

A N. meningitidis mutant deleted of the *gshB* gene was constructed by transformation with a 2-kb DNA fragment containing the *ΔgshB*::*spc* construct amplified from pHT1058 (33), and Spc^r^ clones were selected and named HT2120 (Table 1).

### Production of anti-Cbp protein rabbit antiserum.

A 768-bp DNA fragment devoid of the N-terminal putative hydrophobic domain of Cbp (18 amino acids) was amplified with a set of primers (cbp-1(BamHI) and cbp-2(HindIII)) from NIID84 chromosomal DNA by Prime STAR Max polymerase. The DNA fragment was digested with BamHI and HindIII and was cloned into the same cutting sites of expression vector pET24a (Invitrogen), resulting in pHT1376. The plasmid pHT1376 was transformed into E. coli strain BL21(DE3) (NEB), and the transformant was cultured in 150 ml MagicMedia (Invitrogen) at 30°C overnight with shaking. The subsequent purification of a recombinant protein and generation of polyclonal rabbit serum to the putative hydrophilic domain of the Cbp protein were performed as described previously (81).

### RT-PCR.

Bacteria grown on GC agar plates were suspended in 20 ml of GC to an OD_600_ of 0.1 and continuously cultured to mid-log phase (OD_600_ of ∼0.6) at 37°C with shaking. The total RNA was isolated from the harvested bacteria using a FastGene RNA Premium kit (Nihon Genetics, Japan). RT-PCR was performed using a SuperScript III One-Step RT-PCR system with Platinum *Taq* DNA polymerase (Invitrogen) and approximately 0.1 μg of total RNA according to the manufacturer’s instructions. Reverse transcriptase reactions (50°C for 30 min) were omitted (-RT in Fig. S2 in the supplemental material) to confirm that the amplicon was derived from RNA. The products were visualized by electrophoresis in a 2% agarose gel followed by ethidium bromide staining.

### Tissue culture.

HBMEC were cultivated as described previously (63). In each experiment, HBMEC were seeded in a culture flask or dish or on a cover glass and were used within 2 days of reaching confluence.

### Assessment of host cell-associated and internalized bacteria.

HBMEC were cultivated on gelatin-coated 96-well tissue culture plates (Iwaki, Japan) at 37°C for 2 days in a 5% CO_2_ atmosphere to a concentration of 1 × 10^4^ cells/well. Two hours prior to bacterial infection, the culture medium was replaced with assay medium (AM), which was MCDB131 (Invitrogen) supplemented with 10% fetal bovine serum (FBS), 90 μg ml^−1^ heparin, and 3 mM glutamine. The bacterial suspension was prepared in AM at an OD_600_ of 0.05, which corresponded to approximately 5 × 10^6^ CFU/100 μl. The multiplicity of infection (MOI) was 500, a condition previously used in other studies (45, 83), to examine efficient N. meningitidis internalization (33, 42). A 100-μl portion of the bacterial suspension was inoculated onto host cell monolayers in duplicate for each assay, and the reaction mixtures were incubated at 37°C for 4 h in a 5% CO_2_ atmosphere. In order to evaluate bacterial adherence, the monolayers were washed with prewarmed AM four times to remove nonadherent bacteria. Adherent bacteria were released by the addition of phosphate-buffered saline (PBS) containing 2% saponin, and CFU levels were assessed on GC agar plates with appropriate dilutions to count cell-adherent bacteria. In order to evaluate internalized bacteria, AM containing 150 μg ml^−1^ of gentamicin was added, and cultures were incubated for 1 h in order to kill all extracellular bacteria. Almost all (>99.999%) of up to 5 × 10^7^ extracellular meningococci were killed under these experimental conditions (data not shown). The amounts of internalized bacteria that were not killed by the gentamicin treatment were assessed by the addition of PBS containing 2% saponin and by plating on GC agar after appropriate dilutions were made to count bacterial numbers as CFU levels. Results are expressed as means ± standard deviations (SD), and bacterial numbers were statistically compared using the two-tailed Student's *t* test.

### Western blotting.

N. meningitidis, grown on GC agar plates, was suspended in PBS. Bacteria were harvested at an OD_600_ of 20 and resuspended in 1 ml 1× SDS buffer and boiled for 10 min. SDS-PAGE and Western blotting were performed as described previously (38).

### Bacterial l-cysteine uptake assay.

The l-cysteine transport assay was performed as follows. N. meningitidis strains grown overnight on GC agar plates at 37°C in 5% CO_2_ were suspended in 2 ml buffer A (50 mM potassium buffer [pH 7.0] and 0.5 mM MgCl_2_) (31, 32) and harvested by centrifugation at 10,000 × *g* at 4°C for 2 min. The bacterial pellet was resuspended in 2 ml buffer A and centrifuged again. These procedures were repeated 3 times to wash bacteria. The resultant pellets were suspended in 1 ml buffer A to adjust the bacterial concentration at an OD_600_ of 10. Ten microliters of 300 μM l-[^35^S]cysteine (America Radiolabeled Chemicals) (specific activity, 1,075 Ci/mmol) was mixed with 10 μl of a 3-fold-higher concentration of SM containing 180 mM NaCl under standard conditions. A 3-fold concentration of l-[^35^S]cysteine was added to the reaction mixture when substrate dependency for CTS was examined (Fig. 3C; see also Fig. S3A). The mixture was prewarmed at 37°C for 5 min. Assays were initiated by the addition of 10 μl of the bacterial suspension and was incubated at 37°C for 5 min. Further incubations were performed to optimize the incubation time (Fig. 3B; see also Fig. S3B). The reaction was terminated by the removal of substrates by subsequent maximal centrifugation at 4°C for 1 min. The resultant pellet (bacteria) was washed 3 times with 0.5 ml PBS containing 2% saponin. The resultant pellet was resuspended in 50 μl buffer A. Radioactivity was assessed by scintillation counting in an 8-ml vial containing 5 ml Filtron-X (National Diagnostics, USA). The nonspecific binding of l-[^35^S]cysteine to bacteria was assessed in control bacterial samples that were inactivated at 70°C for 30 min, and the value obtained was subtracted from all experimental values. Protein concentrations were measured using a bicinchoninic acid (BCA) protein assay kit (Thermo), with bovine serum albumin (BSA) used as a standard. l-Cysteine transport values were expressed as means ± SD representing nanomoles of substrate transported per minute per milligram of bacterial protein.

### Observations of meningococci and ezrin accumulation by immunofluorescence staining.

HBMEC monolayers, grown on a cover glass at 37°C in a 5% CO_2_ atmosphere for 2 days, were infected with N. meningitidis in AM at an MOI of 500 for 4 h. The infected HBMEC monolayers were washed four times with 500 μl prewarmed AM, fixed with 4% paraformaldehyde–PBS for 15 min, permeabilized with 0.2% Triton X-100–PBS, and blocked with 2% BSA–PBS for 30 min. The resultant monolayers were incubated with the anti-ezrin monoclonal 3C12 antibody (Santa Cruz, USA) and diluted 100-fold for 45 min and then with the Alexa Fluor 488-conjugated F(ab´) fragment of anti-mouse IgG (Invitrogen) and then diluted 200-fold for 45 min under moist and dark conditions. Glass coverslips were mounted on a glass slide with ProLong Gold antifade reagent (Invitrogen). Infected HBMEC and the attached meningococci were observed using a BX51 microscope with a 100× oil immersion objective.

### Monitoring meningococcal survival in HBMEC.

A meningococcal intracellular survival assay in HBMEC was performed as follows. Confluent HBMEC monolayers seeded on gelatin-coated 24-well tissue culture plates (Iwaki) were infected with meningococci at an MOI of 5,000 for 2 h in order to obtain the maximal amount of internalized bacteria (32). A high dosage of meningococci did not exert any cytotoxic effect on HBMEC (data not shown). Monolayers were washed with AM four times and then incubated with AM containing 150 μg ml^−1^ gentamicin for 1 h in order to kill extracellular bacteria. Gentamicin-containing AM was replaced with AM, and the reaction mixtures were incubated further at 37°C in 5% CO_2_ to monitor the number of bacteria in HBMEC. This time point was defined as time zero. Intracellular bacteria were measured at time zero and after 1, 2, 3, and 4 h, and bacterial numbers were counted as CFU by plating on GC agar plates after appropriate dilutions. Survival percentages were calculated by the following formula: 100 × (CFU at the indicated hour[s]))/(CFU at time zero). The results were expressed as means ± SD.

### Measurement of glutathione concentrations in bacteria only or bacteria that infected HBMEC.

N. meningitidis strains grown on GC agar plates at 37°C overnight in 5% CO_2_ were suspended in 15 ml AM or GCB in order to adjust the bacterial concentration to an OD_600_ of 0.15/ml and were then incubated at 37°C for 4 h in 5% CO_2_, or bacteria were used to infect HBMEC seeded on a 150-mm-diameter dish at an MOI of 500 for 4 h. Bacterial samples were prepared as described previously (33). In brief, harvested bacteria were suspended in 1 ml H_2_O and were centrifuged at 10,000 × *g* at 4°C for 2 min. This procedure was repeated 3 times. The resultant pellets were suspended in 100 μl of 0.5% sulfosalicylic acid, and bacteria were lysed using three freeze/thaw cycles, briefly sonicated, and centrifuged again at 14,000 × *g* at 4°C for 10 min. The glutathione concentration in the supernatant was measured with a total glutathione quantification kit (Dojindo, Japan). Protein concentrations were measured with a BCA protein assay kit. Glutathione concentrations in bacteria were expressed as means ± SD of nanomoles of glutathione per milligram of bacterial protein.

### Assay of sensitivity to H_2_O_2_ and paraquat *in vitro*.

N. meningitidis strains grown on GC agar plates at 37°C overnight in 5% CO_2_ were suspended in 1.5 ml GCB, and the bacterial suspension was prepared in 1 ml GCB at an OD_600_ of 0.01, which corresponded to 1 × 10^7^ bacteria/ml. The indicated concentration of H_2_O_2_ (0.25, 0.5, or 1 mM) or paraquat (2, 5, or 50 mM), which represents the conditions normally applied to *in vitro* neisserial analyses (51, 84–87), was added to 1 ml of the bacterial suspension and incubated at 37°C for 15 min (for H_2_O_2_) or for 60 min (for paraquat) with gentle shaking. Surviving bacteria were counted as CFU by plating on GC agar plates after appropriate dilutions. The CFU of bacteria not treated with H_2_O_2_ or paraquat at time zero was defined as 100%, and the survival rate was calculated using the following formula: 100 × (CFU treated with H_2_O_2_ or paraquat)/(CFU not treated with H_2_O_2_ or paraquat).

### Monitoring of N. meningitidis growth in liquid media.

N. meningitidis strains grown on GC agar plates at 37°C overnight in 5% CO_2_ were suspended in 10 ml buffer A and centrifuged at 7,000 × *g* for 2 min. The bacterial pellet was resuspended in 10 ml buffer A and centrifuged again. This procedure was repeated 3 times. The resultant pellets were suspended in 5 ml buffer A. Bacterial suspensions were prepared at an OD_600_ of 0.1 in 10 ml GCB or SM supplemented with the indicated concentration of cysteine. Since the cysteine solution was made from cysteine HCl salt, the same amount and concentration of NaOH solution were added for neutralization. Bacteria were cultivated at 37°C with shaking at a rate of 160 rpm/min. Meningococcal growth was monitored as OD_600_ by the use of a SmartSpec Plus spectrophotometer (Bio-Rad).

### Statistical analyses.

Results are expressed as means ± SD. Results of determinations of adhered and internalized bacterial numbers, ratios of internalized/adhered bacteria, glutathione measurements, l-cysteine uptake measurements, and meningococcal survival measurements in HBMEC were compared using the two-tailed Student's *t* test, and *P* values of <0.05 were considered to be significant.
