# Supplementary figures and images for: The Meningococcal Cysteine Transport System Plays a Crucial Role in Neisseria meningitidis Survival in Human Brain Microvascular Endothelial Cells
Source: mBio. 2018 Dec 11;9(6):e02332-18. doi: 10.1128/mBio.02332-18 (PMC6299482; doi:10.1128/mBio.02332-18)

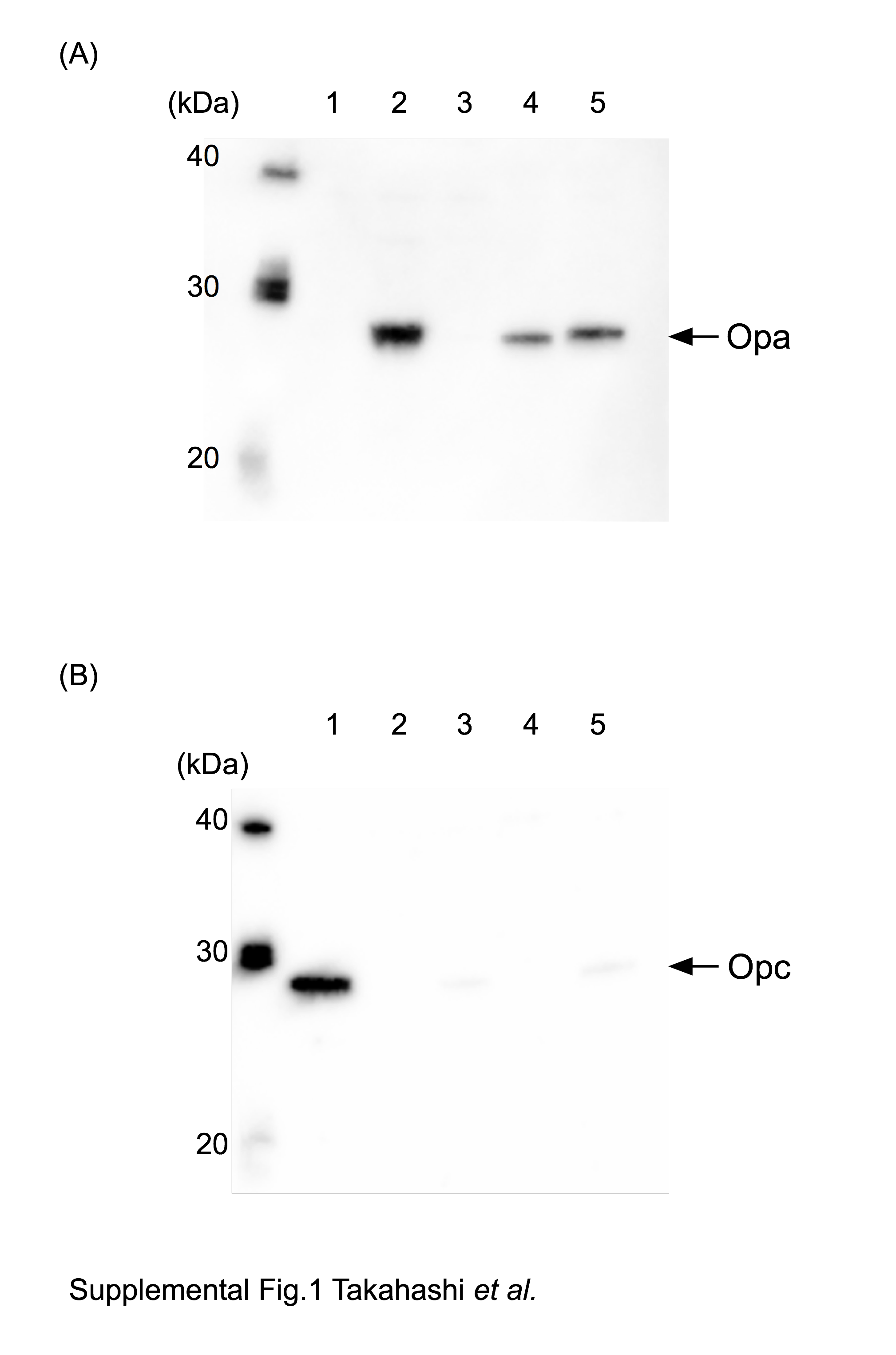

Supplement: Fig S1 [file mbo006184207sf1.tif]

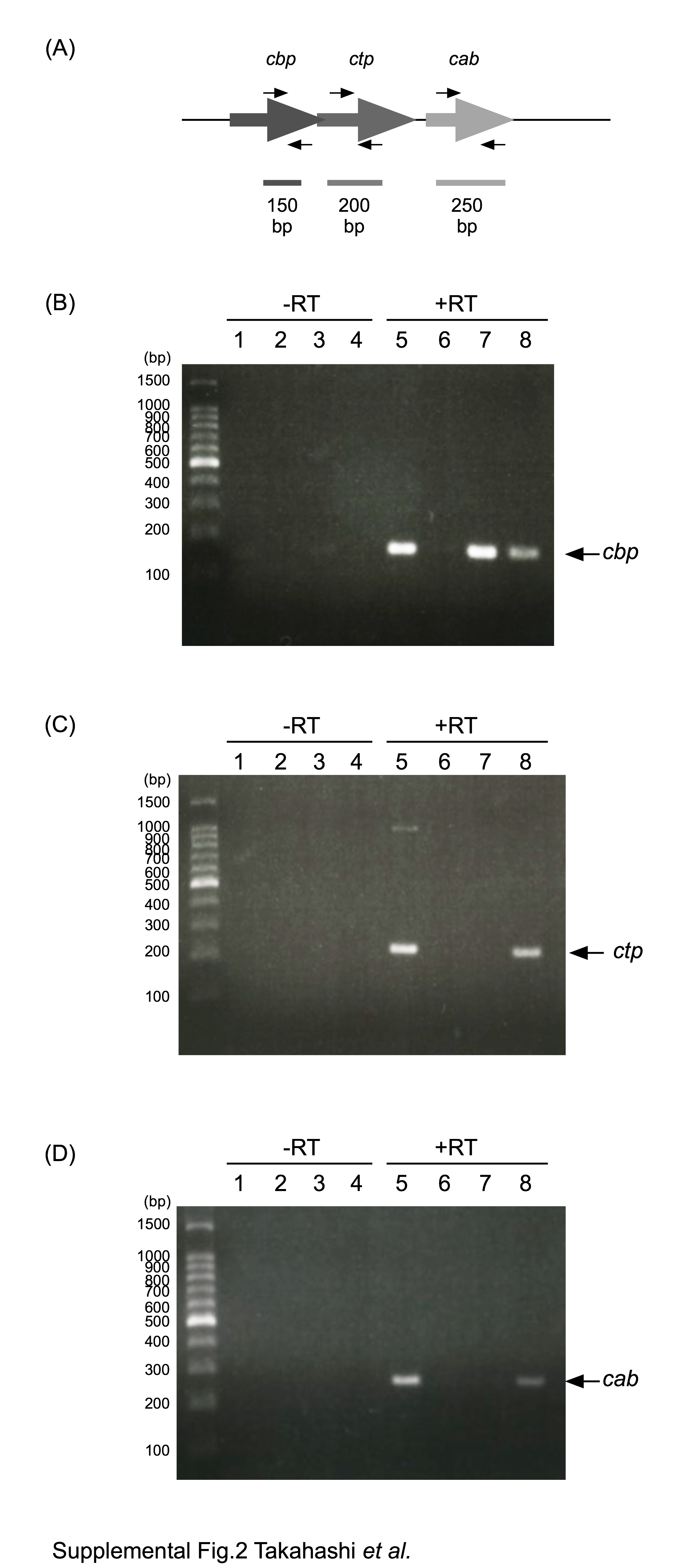

Supplement: Fig S2 [file mbo006184207sf2.tif]

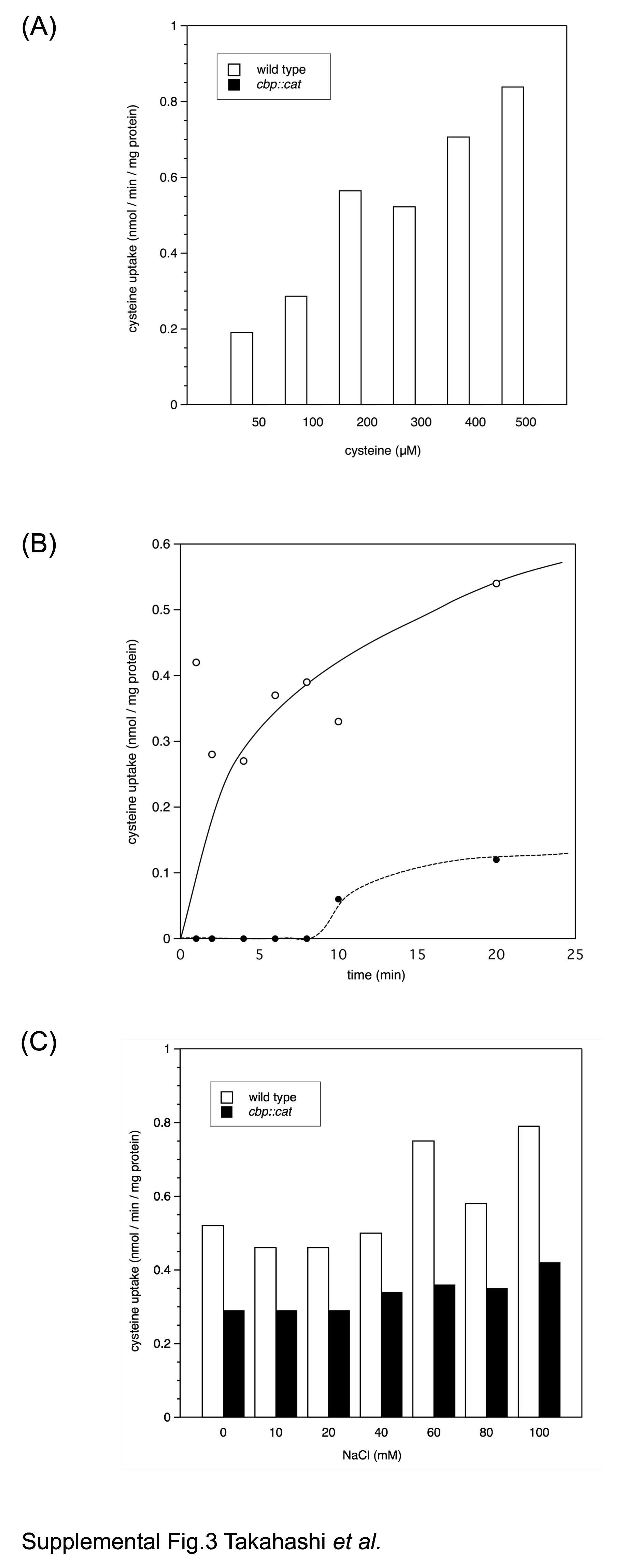

Supplement: Fig S3 [file mbo006184207sf3.tif]

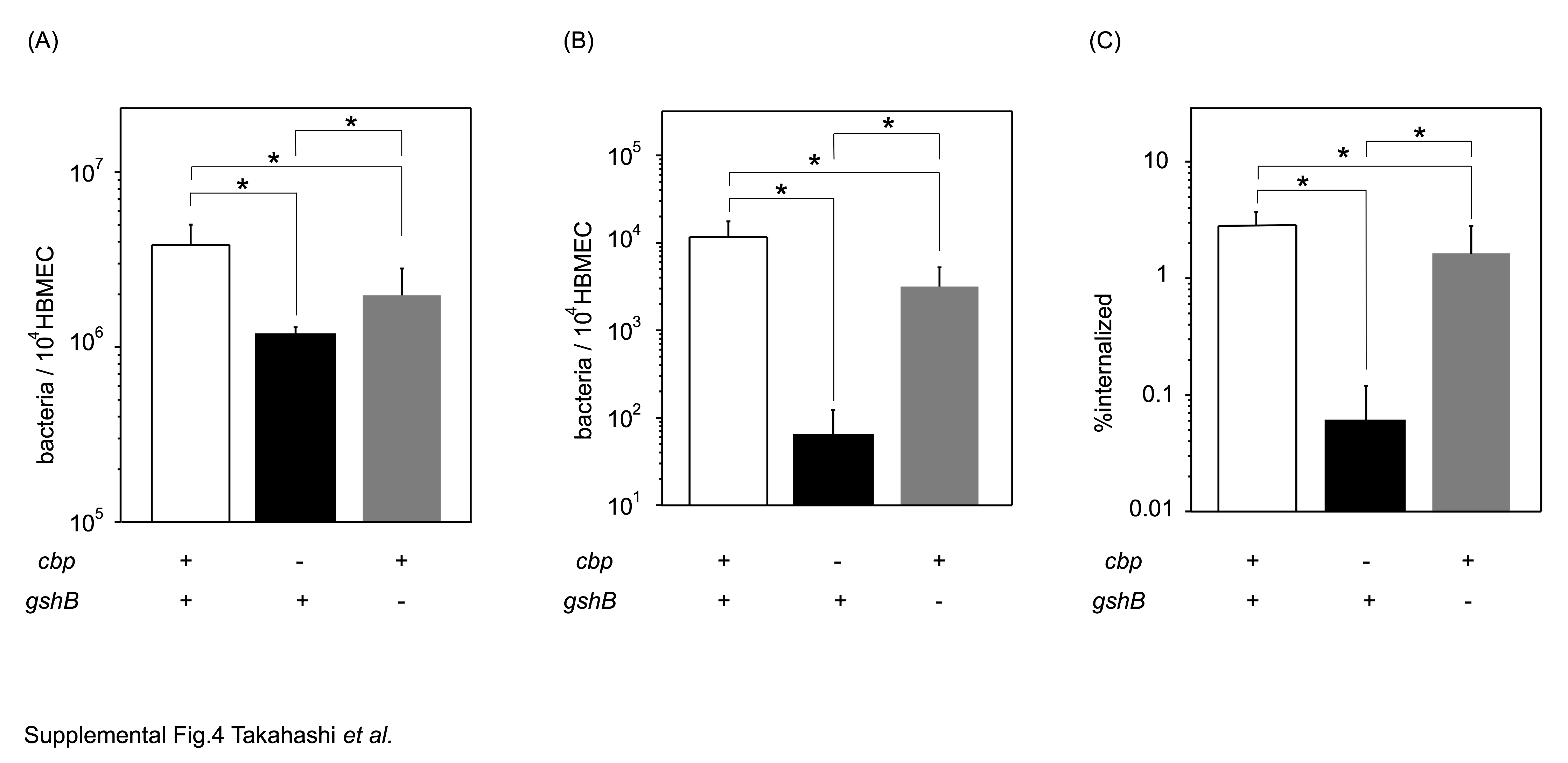

Supplement: Fig S4 [file mbo006184207sf4.tif]

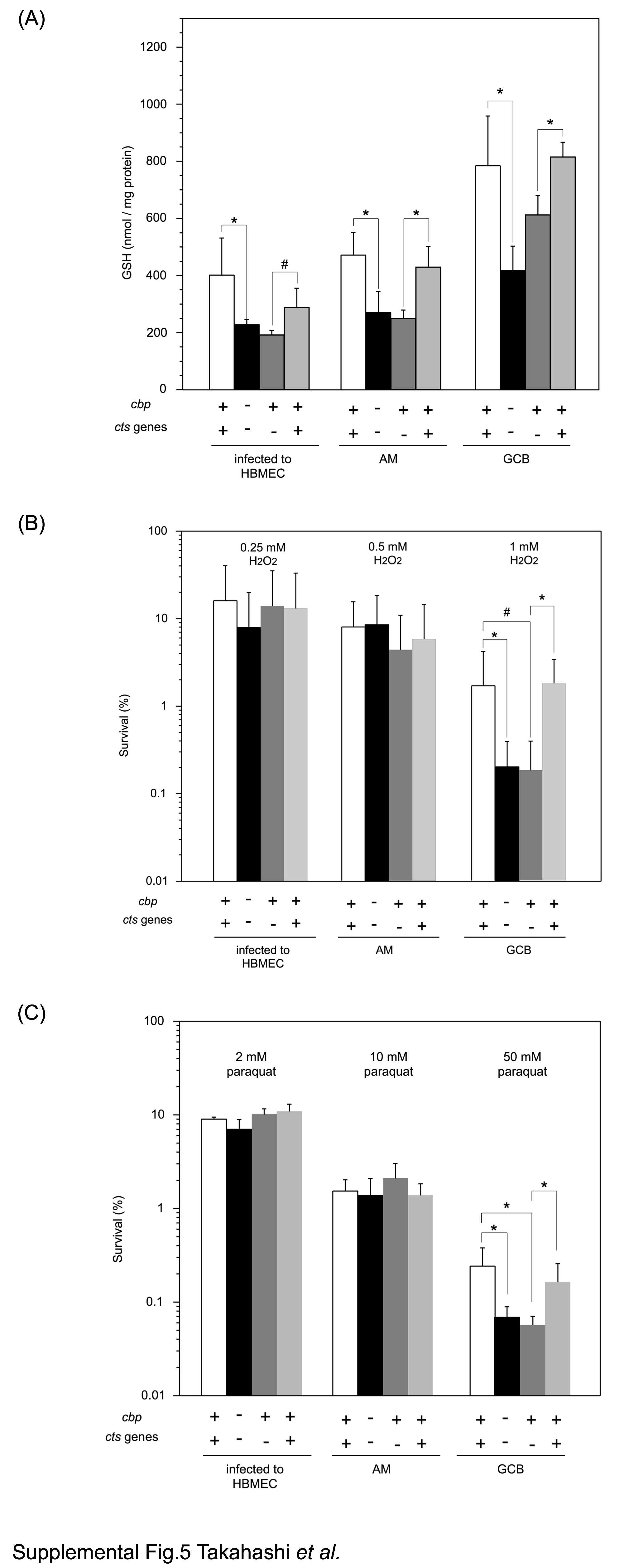

Supplement: Fig S5 [file mbo006184207sf5.tif]

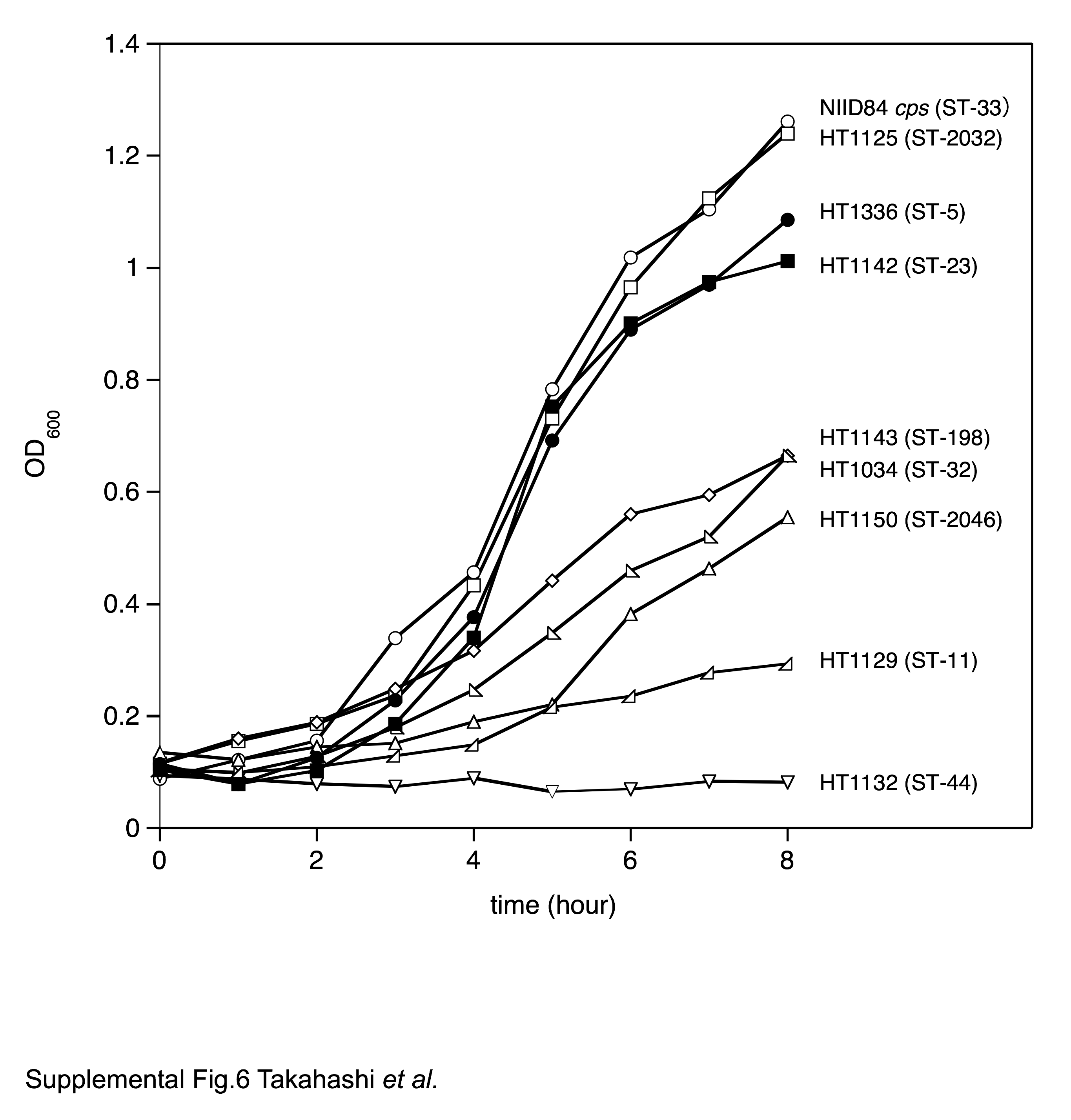

Supplement: Fig S6 [file mbo006184207sf6.tif]
