# Supplementary material for: The Meningococcal Cysteine Transport System Plays a Crucial Role in Neisseria meningitidis Survival in Human Brain Microvascular Endothelial Cells
Source: mBio. 2018 Dec 11;9(6):e02332-18. doi: 10.1128/mBio.02332-18 (PMC6299482; doi:10.1128/mBio.02332-18)
Supplement: TABLE S1 [file mbo006184207st1.docx]

**Supplemental Table 1. Oligonucleotides used in this study**

Primer target gene (vector) Nucleotide sequence (5´-3´) Reference

cbp-1 *cbp* ATGTTGAAAAAATTCGTACTC This study

cbp-2 *cbp* TTATTGAACACTGATGTCTTT This study

cbp-3 *cbp* GGAGCGGTGCCGTATTGGTTG This study

cbp-4 *cbp* AGCTGTAAGGATCGGATTTGT This study

pTTQ-1-cbp-5 *cbp* ACACAGGAAACAGCGATGTTGAAAAAATTCGTACTC This study

M13-47-cbp-6 *cbp* TTTCCCAGTCACGACTTATTTGGCTGCGCCTTCATT This study

pTTQ-2(PO+RBS) pTTQ18 CGCTGTTTCCTGTGTGAAATT This study

M13-47 reverse pTTQ18 GTCGTGACTGGGAAAACCCTGGCG This study

pTTQ-5 pTTQ18 AGGCGATTAAGTTGGGTAACGCCA This study

pTTQ-6 pTTQ18 TGCAGCACATCCCCCTTTCGCCAG This study

pTTQ-5’(15mer) -M13-RV-Long pTTQ18 CCAACTTAATCGCCTAATTTCACACAGGAAACAGCTATGACCATG This study

pTTQ-6’(15mer) -M13-47-Long pTTQ18 GGGGGATGTGCTGCACGCCAGGGTTTTCCCAGTCACGACGTTGTA This study

ggt-5’(15mer)-ptac-13 pTTQ18 ATTTTCACCCAACCCCGCGCGTTGGCCGATTCATTA This study

ggt-3’(15mer)-ptac-14 pTTQ18 ATCATACCCCACCCCCGGCATCAGAGCAGATTGTACTGA This study

ggt-1 *ggt* ATGCCCTTGTATGGATCA (1)

ggt-2 *ggt* CTAATCACCCATCACTCGACCT (1)

cts operon-1 *cts* CGTATGTCCGAATGTTGCCCAGGCTTTGCT

cts operon-2 *cts* TGGAAAAAACCGGCCACAGCTTTATCAAAT This study

M13-47’(15mer) -pMW-down-1 pMW119 GGGAAAACCCTGGCGGGAAAACCCTGGCGTTACCCAACT  This study

M13-RV’(15mer)-pMW-down-2 pMW119 CATAGCTGTTTCCTGCAGTCACGACGTTGTAAAACGACG This study

M13-RV CAGGAAACAGCTATGAC TakaraBio

M13-47 CGCCAGGGTTTTCCCAGTCACGAC TakaraBio

ggt-5’(15mer)-pMW119-F pMW119 ATCATACCCCACCCGCTTATCGAATCAAAGCTGCCGAC  This study

ggt-3’(15mer)-pMW119-R pMW119 ATTTTCACCCAACCCTTTATGCTTCCGGCTCGTATG   This study

cbp-1(BamHI) *cbp* CG**GGATCC**GGCGGTTCGGAAGGCGGCAGC This study

cbp-2(HindIII) *cbp* CCC**AAGCTT**TTGAACACTGATGTCTTTTCC This study

*The nucleotides shown in underlined indicated the 15 mer nucleotides originally added for cloning by SLiCE (2).

** The nucleotides shown in double-underlined letter indicated the mutually complemented 15 mer nucleotides for the nucleotide sequence shown in underlined.

# The nucleotides shown in bold case letter indicated the restriction enzyme sites for BamHI and HindIII, respectively.

1. Takahashi H, Tanaka H, Inouye H, Kuroki T, Watanabe Y, Yamai S, Watanabe H. 2002. Isolation from a healthy carrier and characterization of a Neisseria meningitidis strain that is deficient in gamma-Glutamyl aminopeptidase activity. J Clin Microbiol 40:3035-7.

2. Motohashi K. 2015. A simple and efficient seamless DNA cloning method using SLiCE from Escherichia coli laboratory strains and its application to SLiP site-directed mutagenesis. BMC Biotechnol 15:47.
