# Supplementary material for: The Meningococcal Cysteine Transport System Plays a Crucial Role in Neisseria meningitidis Survival in Human Brain Microvascular Endothelial Cells
Source: mBio. 2018 Dec 11;9(6):e02332-18. doi: 10.1128/mBio.02332-18 (PMC6299482; doi:10.1128/mBio.02332-18)
Supplement: TABLE S2 [file mbo006184207st2.docx]

Supplemental Table 2

**Concentration of synthetic medium used in this study**

(A) Neisserial chemically defined medium (NCDM)*

mg/L mM

KCl 186 2.5

NH_4_Cl 400 7.5

Na_2_HPO_4_ 1065 7.5

KH_2_PO_4_ 170 1.25

Sodium citrate 2H_2_O 650 2.2

**MgSO_4_ 7 H_2_O 61.6 0.25**

**MnSO_4_ 11.3 0.075**

L-arginine-HCl 12 57

L-glycine 50 670

L-glutamate Na 5.5 29

Glucose 5000 27.8

(B) 1/4 volume of MCDB131 devoid of cysteine and NaCl

(Referred from Thermo Fisher Scientific)

Amino Acids mg/L mM

Glycine 0.575 0.00766

L-Alanine 0.675 0.00757

L-Arginine hydrochloride 15.8 0.075

L-Asparagine-H_2_O 28.75 0.025

L-Aspartic acid 3.3 0.25

L-Glutamic Acid 1.1 0.0075

L-Histidine hydrochloride-H_2_O 10.5 0.05

L-Isoleucine 16.5 0.126

L-Leucine 32.8 0.25

L-Lysine hydrochloride 45.5 0.248

**L-Methionine 3.75 0.025**

L-Phenylalanine 8.25 0.05

L-Proline 2.75 0.025

L-Serine 8.0 0.0762

L-Threonine 3.0 0.0252

L-Tryptophan 1.025 0.005

L-Tyrosine 4.525 0.025

L-Valine 29.25 0.25

Vitamins mg/L mM

Biotin 0.0018 7.5 E-6

Choline chloride 3.0 0.00629

Folinic Acid Calcium salt 0.15 0.00029

Niacinamide 1.525 0.0125

Pyridoxine hydrochloride 0.525 0.00255

Riboflavin 0.00095 2.53E-6

Thiamine hydrochloride 0.85 0.002522

Vitamin B12 0.0034 2.509E-6

i-Inositol 1.8 0.01

Inorganic Salts mg/L mM

(NH_4_)_6_Mo_7_O_24_-4H_2_O 0.000925 7.5E-7

NH_4_VO_3_ 1.5E-4 1.23E-6

CaCl_2_-2H_2_O 58.75 0.4

**CuSO_4_-5H_2_O 0.0003 1.2E-6**

**FeSO_4_-7H_2_O 0.07075 0.0002544964**

**MgSO_4_-7H_2_O 616.0 2.5**

**MnSO_4_-H_2_O 5.0E-5 0.295857975E-7**

NiCl_2_ 6H_2_O 1.8E-5 7.45798375E-8

KCl 74.5 1

H_2_SeO_3_ 0.00095 7.3643415E-6

NaHCO_3_) 294.0 3.5

Na_2_SiO_3_ 9H_2_O 0.7 0.00246478875

Na_2_HPO_4_-7H_2_O 33.5 0.125

**ZnSO_4_-H_2_O 7.5E-5 2.60416675E-7**

Other Components mg/L mM

Adenine 0.03375 0.00025

D-Glucose 250.0 1.388888825

Lipoic Acid 0.000525 2.5485435E-6

Phenol Red 3.1 0.00823591925

Putrescine 2HCl 5.0E-5 3.10559E-7

Sodium Pyruvate 2.5 0.25

Thymidine 0.006 2.479339E-5

*The composition was same as described (1)

**The composition was referred to the data provided by Thermo Fisher Scientific (<https://www.thermofisher.com/order/catalog/product/10372019>).

***The material shown in bold indicated the compounds containing sulfur.

1. Catlin BW. 1973. Nutritional profiles of *Neisseria gonorrhoeae*, *Neisseria meningitidis*, and *Neisseria lactamica* in chemically defined media and the use of growth requirements for gonococcal typing. J Infect DIs 128:178-94.
